# *In silico* Assessment of Pharmacotherapy for Human Atrial Patho-Electrophysiology Associated With hERG-Linked Short QT Syndrome

**DOI:** 10.3389/fphys.2018.01888

**Published:** 2019-01-11

**Authors:** Dominic G. Whittaker, Jules C. Hancox, Henggui Zhang

**Affiliations:** ^1^Faculty of Biological Sciences, School of Biomedical Sciences, University of Leeds, Leeds, United Kingdom; ^2^Biological Physics Group, School of Physics and Astronomy, The University of Manchester, Manchester, United Kingdom; ^3^Cardiovascular Research Laboratories, Department of Physiology, Pharmacology and Neuroscience, School of Medical Sciences, University of Bristol, Bristol, United Kingdom; ^4^School of Computer Science and Technology, Harbin Institute of Technology, Harbin, China; ^5^Space Institute of Southern China, Shenzhen, China; ^6^Key Laboratory of Medical Electrophysiology of Ministry of Education and Medical Electrophysiological Key Laboratory of Sichuan Province, Institute of Cardiovascular Research, Southwest Medical University, Luzhou, China

**Keywords:** arrhythmia, short QT syndrome, atrial fibrillation, hERG, class I anti-arrhythmics, human atria, potassium channels

## Abstract

Short QT syndrome variant 1 (SQT1) arises due to gain-of-function mutations to the *human Ether-à-go-go-Related Gene* (*hERG*), which encodes the α subunit of channels carrying rapid delayed rectifier potassium current, *I*_Kr_. In addition to QT interval shortening and ventricular arrhythmias, SQT1 is associated with increased risk of atrial fibrillation (AF), which is often the only clinical presentation. However, the underlying basis of AF and its pharmacological treatment remain incompletely understood in the context of SQT1. In this study, computational modeling was used to investigate mechanisms of human atrial arrhythmogenesis consequent to a SQT1 mutation, as well as pharmacotherapeutic effects of selected class I drugs–disopyramide, quinidine, and propafenone. A Markov chain formulation describing wild type (WT) and N588K-hERG mutant *I*_Kr_ was incorporated into a contemporary human atrial action potential (AP) model, which was integrated into one-dimensional (1D) tissue strands, idealized 2D sheets, and a 3D heterogeneous, anatomical human atria model. Multi-channel pharmacological effects of disopyramide, quinidine, and propafenone, including binding kinetics for *I*_Kr_/hERG and sodium current, *I*_Na_, were considered. Heterozygous and homozygous formulations of the N588K-hERG mutation shortened the AP duration (APD) by 53 and 86 ms, respectively, which abbreviated the effective refractory period (ERP) and excitation wavelength in tissue, increasing the lifespan and dominant frequency (DF) of scroll waves in the 3D anatomical human atria. At the concentrations tested in this study, quinidine most effectively prolonged the APD and ERP in the setting of SQT1, followed by disopyramide and propafenone. In 2D simulations, disopyramide and quinidine promoted re-entry termination by increasing the re-entry wavelength, whereas propafenone induced secondary waves which destabilized the re-entrant circuit. In 3D simulations, the DF of re-entry was reduced in a dose-dependent manner for disopyramide and quinidine, and propafenone to a lesser extent. All of the anti-arrhythmic agents promoted pharmacological conversion, most frequently terminating re-entry in the order quinidine > propafenone = disopyramide. Our findings provide further insight into mechanisms of SQT1-related AF and a rational basis for the pursuit of combined *I*_Kr_ and *I*_Na_ block based pharmacological strategies in the treatment of SQT1-linked AF.

## Introduction

The short QT syndrome (SQTS) is characterized primarily by a short QT interval on the ECG, which corresponds to abbreviated ventricular repolarisation. However, symptomatic atrial fibrillation (AF) has also been reported as a common first clinical presentation of the SQTS (Schimpf et al., 2005), suggesting that pathophysiological mechanisms leading to shortening of the QT interval also affect the atria, which can increase susceptibility to AF. The N588K mutation to the *human Ether-à-go-go-Related gene (hERG)*, which encodes the α subunit of channels carrying rapid delayed rectifier potassium current, *I*_Kr_, underlies a form of SQTS variant 1 (SQT1) (Brugada et al., 2004), and has been associated with a high incidence of AF in affected probands—as high as 50% (Hu et al., 2017). Whilst ventricular arrhythmia substrates in SQT1 have received much attention (Zhang and Hancox, 2004; Patel and Antzelevitch, 2008; Adeniran et al., 2011), there have been comparatively fewer studies investigating mechanisms by which SQT1 mutations promote AF, which can be an important biomarker of the SQTS. Furthermore, effective management of AF remains a challenge, and is incompletely understood in the context of SQT1 (Enriquez et al., 2016; Hancox et al., 2018).

A previous simulation study (Loewe et al., 2014b) used the Courtemanche-Ramirez-Nattel (CRN) mathematical model of the human atrial action potential (AP) (Courtemanche et al., 1998) to demonstrate shortening of the atrial effective refractory period (ERP) and tissue excitation wavelength (WL) consequent to the N588K-hERG mutation. This was shown to facilitate initiation and sustenance of spiral waves in idealized two-dimensional (2D) sheets of human atrial tissue, which is a likely mechanism for increased susceptibility to AF. However, both the intrinsic electrical heterogeneities and complex anatomy of the human atria have also been suggested to influence arrhythmia vulnerability and dynamics in overall response to K^+^ channel mutations (Colman et al., 2017; Whittaker et al., 2017b). Consequently, the first aim of the present study was to assess the arrhythmogenicity of the N588K-hERG mutation in anatomically-detailed models of the human atria with realistic structure and inclusion of regional differences in electrophysiology.

The class Ia anti-arrhythmic drug quinidine is typically used as the frontline therapy for QT interval normalization in SQT1 (Gaita et al., 2004; Giustetto, 2006; Hu et al., 2017), and disopyramide has been suggested as a possible alternative (Dumaine and Antzelevitch, 2006; Schimpf et al., 2007). Regarding management of atrial arrhythmias, the class Ic anti-arrhythmic drug propafenone has been reported to be effective at preventing recurrent episodes of (paroxysmal) AF mediated by the N588K mutation to hERG (Hong et al., 2005; Bjerregaard et al., 2006), maintaining 2 patients free of arrhythmia recurrence for >2 years (Hong et al., 2005). In an experimental setting in which a hERG activator was used to approximate SQT1, quinidine (a blocker of *I*_Na_ and *I*_Kr_) was effective at extending atrial AP duration and ERP and preventing AF (Nof et al., 2010), more so than E-4031 (a selective *I*_Kr_ blocker) or lidocaine (an *I*_Na_ blocker) alone, suggesting that K^+^ and Na^+^ channel blocking effects combine synergistically for improved management of AF in SQT1. Similarly, in our previous study, a multi-scale computational modeling approach was used to investigate ventricular pharmacological effects of disopyramide and quinidine in SQT1 (Whittaker et al., 2017a), where combined *I*_Na_ and *I*_Kr_ block by both compounds was shown to prolong ERP to a greater extent than *I*_Na_ or *I*_Kr_ block alone. Mechanisms by which Na^+^ and K^+^-channel blocking agents may provide beneficial effects in the context of SQT1-mediated human atrial pro-arrhythmia remain unclear. A multi-scale cardiac modeling approach is being used increasingly for optimisation of therapy (Yuan et al., 2015). Consequently, the second aim of the present study was to assess the efficacy of the class I drugs disopyramide, quinidine, and propafenone on rate and rhythm control of human atrial arrhythmias mediated by SQT1, from cell to 3D tissue levels, using drug binding models (including reduced potency of drugs against SQT1 mutant *I*_Kr_) with multi-channel pharmacology.

## Methods

### Modeling Human Atrial Electrophysiology

Human atrial electrophysiology was simulated using the Colman et al. model (Colman et al., 2013), as updated recently (Ni et al., 2017), and is hereinafter referred to as the CNZ (Colman-Ni-Zhang) model. The equations for *I*_Kr_ in the CNZ model were replaced with previously developed and validated Markov chain formulations of wild type (WT) and N588K mutant *I*_Kr_/hERG (Whittaker et al., 2017a). The maximal conductance of *I*_Kr_ was set to *g*_Kr_ = 0.0111375$\cdot {\left[K^{+}\right]}_{o}^{0.59}$, where ${\left[K^{+}\right]}_{o}$ is the extracellular potassium concentration (Adeniran et al., 2011), resulting in an action potential duration (APD) at 90% repolarisation (APD_90_) of 248.8 ms at 1 Hz in the baseline human atrial cell model, which is well within the experimentally-measured range of APDs in human atrial myocytes (Bosch et al., 1999; Kim et al., 2002; Dobrev and Ravens, 2003; Katoh et al., 2005; Redpath et al., 2006; Pau et al., 2007). A family of regional human atrial cell models was incorporated into the CNZ model, accounting for distinct differences in electrophysiology of the right and left atrium (RA and LA, respectively), right and left atrial appendages (RAA and LAA, respectively), crista terminalis (CT), pectinate muscles (PM), atrio-ventricular ring (AVR), atrial septum (AS), Bachmann's bundle (BB), and pulmonary veins (PV) (Colman et al., 2013). Changes to maximal ionic conductances relative to the RA model implemented in order to create a family of regional cell models are given in Table S1. The homozygous (N588K) form of SQT1 was modeled as consisting of 100% mutant subunits, whereas the heterozygous (WT-N588K) form was assumed to consist of a 1:1 WT:mutant subunit ratio (Whittaker et al., 2017a).

### Modeling Pharmacological Actions of Disopyramide and Quinidine

In our previous study (Whittaker et al., 2017a), the actions of the class Ia anti-arrhythmic drugs disopyramide and quinidine on human ventricles were simulated in the setting of SQT1. State-dependent binding of disopyramide and quinidine to hERG channels was simulated through addition of drug-bound open and inactivated states to Markov chain formulations of *I*_Kr_, and the guarded receptor model (Starmer et al., 1984) was used to describe use-dependent block of fast sodium current, *I*_Na_, by both agents. Furthermore, the blocking actions of disopyramide and quinidine on slow delayed rectifier potassium current, *I*_Ks_, L-type calcium current, *I*_CaL_, transient outward potassium current, *I*_to_, inward rectifier potassium current, *I*_K1_ (quinidine only), and late sodium current, *I*_NaL_ (quinidine only), were modeled using a simple “pore block” approach. In the present study, the actions of disopyramide and quinidine on human atrial electrophysiology were represented using the same formulations and IC_50_ (half maximal inhibitory concentration) values presented in Whittaker et al. (2017a). In addition, disopyramide and quinidine have both been reported to block the atrial-specific ultra-rapid delayed rectifier potassium current, *I*_Kur_. For disopyramide, the IC_50_ for block of *I*_Kur_ was taken to be 25.0 μM (Aréchiga et al., 2008), and for quinidine the IC_50_ was taken to be 6.6 μM, as measured in human atrial myocytes (Nenov et al., 1998). As the CNZ model does not include late sodium current, quinidine block of *I*_NaL_ was not included in simulations. Full details of disopyramide and quinidine models can be found in Whittaker et al. (2017a).

### Modeling Pharmacological Actions of Propafenone

**Figure 1:**
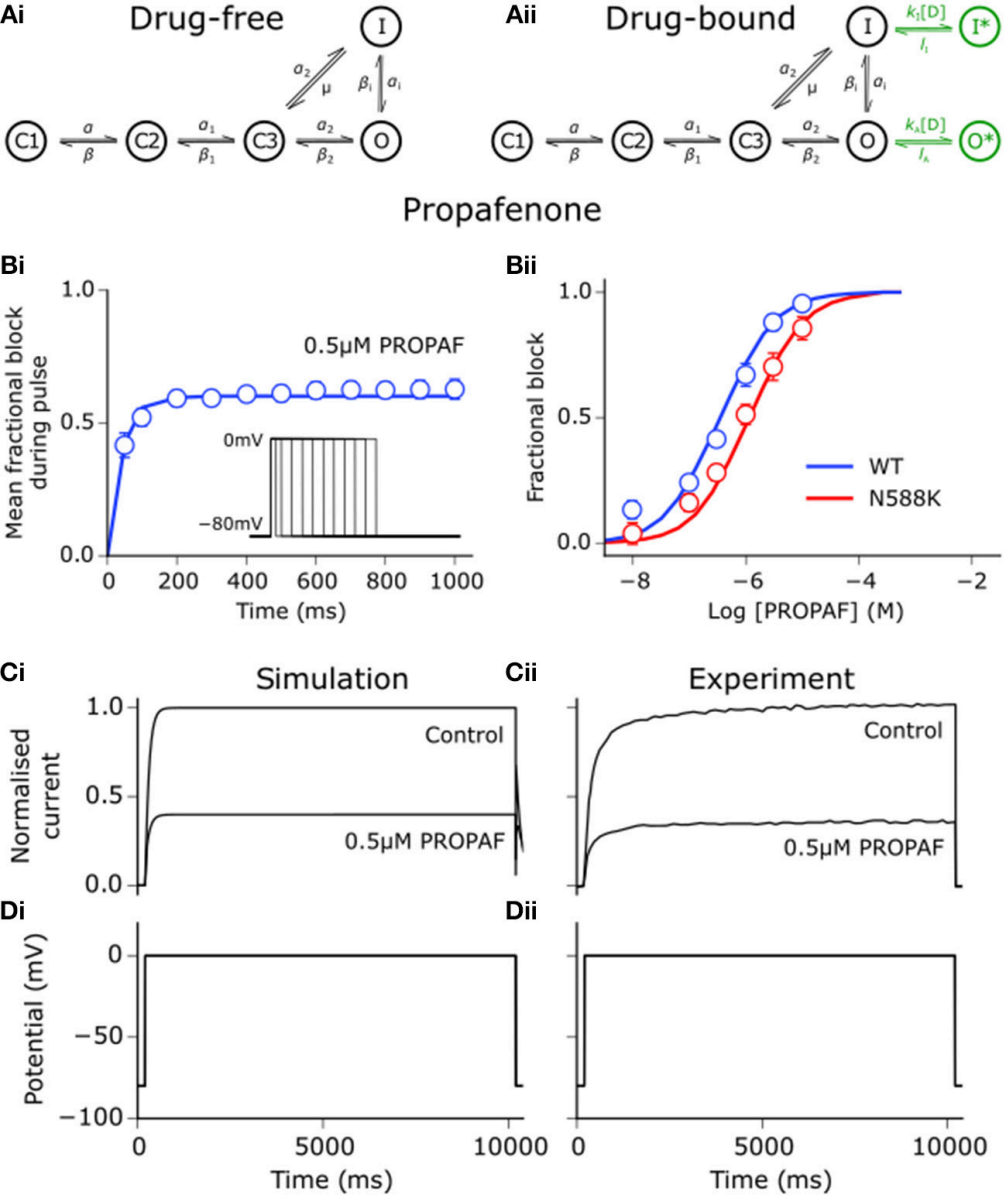
Propafenone interactions with hERG channels. **(Ai)** Drug-free and **(Aii)** drug-bound (additional states shown in green) Markov chain models of *I*_Kr_/hERG. Simulated (solid line) and experimental (points) mean fractional block by 0.5 μM propafenone (PROPAF) of *I*_hERG_ during pulse protocol (shown inset) **(Bi)**, and dose-response curve under WT (blue) and SQT1 mutant N588K (red) conditions **(Bii)**, where IC_50_ values are 390 nM and 936 nM (2.4-fold increase), respectively. **(Ci)** Simulated and **(Cii)** experimental current traces in response to a 10,000 ms depolarising voltage step to 0 mV from a holding potential of −80 mV **(Di,Dii)** under control conditions and with application of 0.5 μM PROPAF – these data were used to validate and not to further train the model. Experimental data at 37°C are taken from Paul et al. (2002) and McPate et al. (2008).

Using the same approach as detailed for disopyramide and quinidine in Whittaker et al. (2017a), state-dependent models of drug block by propafenone were developed. Interactions between propafenone and *I*_Kr_/hERG were developed based on experimental data obtained at 37°C (Paul et al., 2002; McPate et al., 2008), where estimation of parameters for drug-bound states of the Markov chain formulation of *I*_Kr_ was performed using the procedure outlined in Moreno et al. (2016). Binding and unbinding rates to activated and inactivated state channels were allowed to vary freely in order to minimize the difference between simulated and experimental dose-dependent steady state block (WT and N588K), mean fractional block of tail currents during a pulse protocol, and voltage-dependence of tail current block (Paul et al., 2002; McPate et al., 2008). The extended drug-free Markov chain model of *I*_Kr_/hERG is shown in Figure 1, as well as the close concordance between simulated and experimental data regarding propafenone block of *I*_hERG_. Parameters and state affinities are given in Table S2.

**Figure 2:**
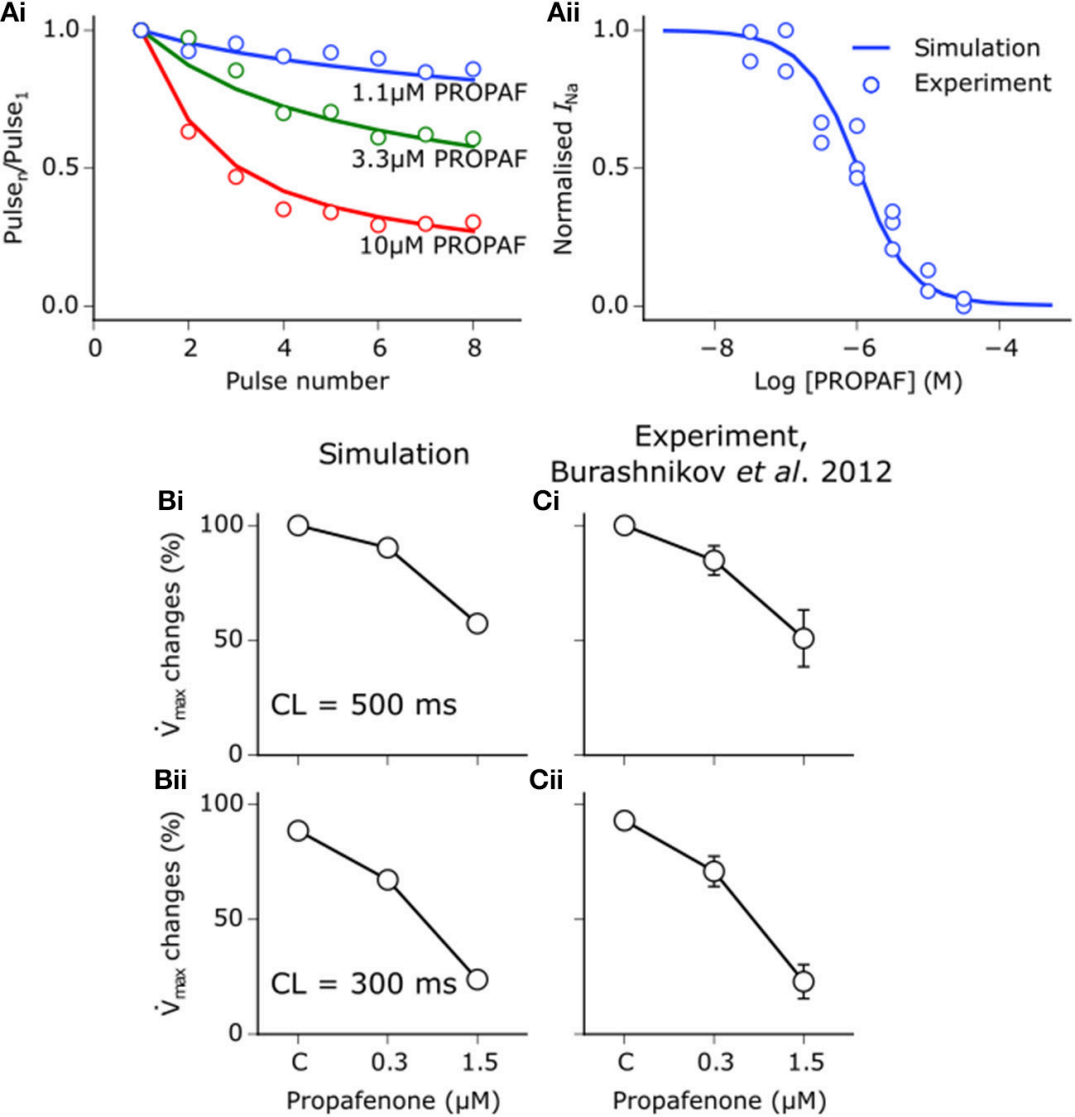
Propafenone interactions with sodium channels. **(Ai)** Simulated (solid lines) and experimental (points) use-dependent block of sodium channels by propafenone (PROPAF) elicited using a series of 8 pulses from −90 mV to 0 mV at 3 Hz for three different test concentrations. **(Aii)** Steady state block of sodium channels using pulses from −90 mV to 0 mV at 3 Hz until a steady state was achieved. Experimental data are taken from Harmer et al. (2008), where the steady state block IC_50_ value is given as 1.4 μM. **(B)** Simulated and **(C)** experimentally-measured PROPAF-induced reduction in maximum upstroke velocity (${\overset{{}^{\circ}}{V}}_{\max}$) upon application of 0.3 and 1.5 μM PROPAF at a cycle length (CL) of (i) 500 ms and (ii) 300 ms. All ${\overset{{}^{\circ}}{V}}_{\max}$ reductions are relative to the control (C) value at a CL of 500 ms. Experimental data, recorded from canine atrial myocytes, are taken from Burashnikov et al. (2012).

Propafenone is a class Ic sodium channel blocking anti-arrhythmic drug (Roden, 2014). Use-dependent block of sodium channels by propafenone was represented using the guarded receptor hypothesis (Starmer et al., 1984), as described for disopyramide and quinidine. Propafenone is predominantly an open state sodium channel blocker with little to no resting state block (Edrich et al., 2005; Burashnikov et al., 2012), and was thus assumed to bind only to activated and inactivated states. Binding and unbinding parameters were constrained based on the dose-dependent, use-dependent, and steady state block of *I*_Na_ by propafenone (Harmer et al., 2008), as shown in Figure 2A. Binding and unbinding parameters and state affinities are given in Table S3.

Whilst propafenone most potently blocks *I*_Kr_ and *I*_Na_, it is a multi-channel blocker, and thus exerts secondary effects on other ion channels, which were modeled using a simple pore block based on IC_50_ values from the literature. Propafenone blocks *I*_CaL_ with an IC_50_ of 1.5–1.7 μM in mammalian cardiac myocytes (Fei et al., 1993; Hancox and Mitcheson, 1997). In human atrial myocytes, propafenone has been shown to block *I*_to_ with an IC_50_ of 4.8 μM (Gross and Castle, 1998; Seki et al., 1999) and *I*_K1_ with an IC_50_ of 16.8 μM (Amorós et al., 2013). Finally, *I*_Kur_ is blocked with an IC_50_ of 4.4 μM (Franqueza et al., 1998). The therapeutic range of propafenone has been estimated to be between 2 and 6 μM (Paul et al., 2002). Taking into account plasma protein binding, estimates of the most likely unbound concentrations have been given as 0.15–0.7 μM (Slawsky and Castle, 1994), 0.2–0.6 μM (Duan et al., 1993), and 0.33–1 μM (Seki et al., 1999). In this study, the effects of three equally-spaced concentrations which fell within this range were studied−0.2, 0.5, and 0.8 μM propafenone. It should be noted that these concentrations are not intended to be compared directly with those chosen for disopyramide and quinidine (1, 2, and 5 μM), which were taken from Whittaker et al. (2017a) and are not equally-spaced. A comparison of IC_50_ values used for disopyramide, quinidine, and propafenone is given in Table S4.

In order to assess whether the combined actions of propafenone in the model induced reasonable rate- and concentration- dependent effects on the AP under control conditions, alterations in the maximum upstroke velocity (${\overset{{}^{\circ}}{V}}_{\max}$) upon application of propafenone were compared with experimental data taken from canine atrial cells (Burashnikov et al., 2012). Figure 2B shows changes in ${\overset{{}^{\circ}}{V}}_{\max}$ upon application of 0.3 and 1.5 μM propafenone at cycle lengths of 500 and 300 ms, where close concordance can be seen between simulation and experiment (Figure 2C). Although experimental data were taken from canine atrial myocytes, the human atrial simulation data nonetheless recapitulated the considerable reduction in ${\overset{{}^{\circ}}{V}}_{\max}$ at both concentrations and cycle lengths, which is primarily due to the sodium channel blocking actions of propafenone.

### Tissue Simulations

Propagation of excitation waves in tissue was described using the monodomain equation,

(1) $$\begin{array}{r} \frac{\partial V}{\partial t}=\nabla \left(\text{D}\nabla V\right)-\frac{I_{\text{ion}}}{C_{\text{m}}}, \end{array}$$

where *V* is the transmembrane voltage, **D** is the global conductivity tensor, *I*_ion_ is the total ionic current, and *C*_m_ is the membrane capacitance. Equation (1) was solved using a finite difference PDE solver based on the explicit forward Euler method and Strang splitting scheme. Effects of the N588K-hERG mutation on ERP, WL, conduction velocity (CV), and spiral wave dynamics were determined using 1D and 2D models of RA tissue, as described previously (Whittaker et al., 2017b, 2018). Spiral waves were initiated in 2D models using an S1-S2 cross-shock protocol, and spiral wave core trajectories over a 5.0 s simulation period were traced by locating phase singularities (Bray and Wikswo, 2002). The behavior of re-entrant excitations in an anatomically-realistic setting was determined using a 3D human atrial structured grid geometry based on the Visible Female dataset (Seemann et al., 2006; Colman et al., 2013; Whittaker et al., 2017b) with rule-based fiber orientations (Krueger et al., 2011). The transverse value of the diffusion coefficient, *D*_⊥_, was set to 0.1 mm^2^ ms^−1^, where an anisotropy ratio (*D*_||_:*D*_⊥_) of 3:1 in directions longitudinal and transverse to fibers was applied in the atrial working myocardium, and a ratio of 9:1 along the fast conducting bundles of the BB, CT, and PM (Colman et al., 2013; Whittaker et al., 2017b), which gave global and specific regional activation times in close agreement with experimental measurements (Lemery et al., 2007). The activation (ACT) time was defined as the time required for the membrane potential to reach −20 mV at each point in the geometry (Ni et al., 2017). Activation-recovery interval (ARI) was defined as the time interval between membrane potential depolarisation to −20 mV and repolarisation to −70 mV (adjusted to −65 mV in the PV region due to higher resting membrane potential) (Ni et al., 2017).

Re-entry was initiated using the phase distribution method (Biktashev and Holden, 1998; Whittaker et al., 2017a), by initiating either a clockwise or anti-clockwise scroll wave (as seen from a RA posterior wall view). Where sustained re-entrant activity was initiated, a power spectrum was obtained through fast Fourier transform analysis of pseudo ECG (pECG) time series (recorded from within the RA cavity). The dominant frequency (DF) was computed in Matlab from the largest peak in the power spectrum density. For simulating the effects of disopyramide, quinidine, and propafenone on re-entrant excitation, state variables for each node within the 3D anatomical model were saved after 2.5 s of a 10.0 s episode of sustained re-entrant activity in drug-free conditions. These were then used as initial conditions for new 3D simulations of duration 7.5 s (giving 10.0 s activity overall) in which varying concentrations of anti-arrhythmic drugs (1, 2, or 5 μM disopyramide and quinidine; 0.2, 0.5, or 0.8 μM propafenone) were applied immediately. This gave 18 simulations in total; 3 doses for each of the 3 anti-arrhythmic drugs, with 2 scroll wave initial conditions (both clockwise and anti-clockwise).

## Results

### Effects of SQT1 Mutant *I*_Kr_ on Human Atrial Action Potentials

**Figure 3:**
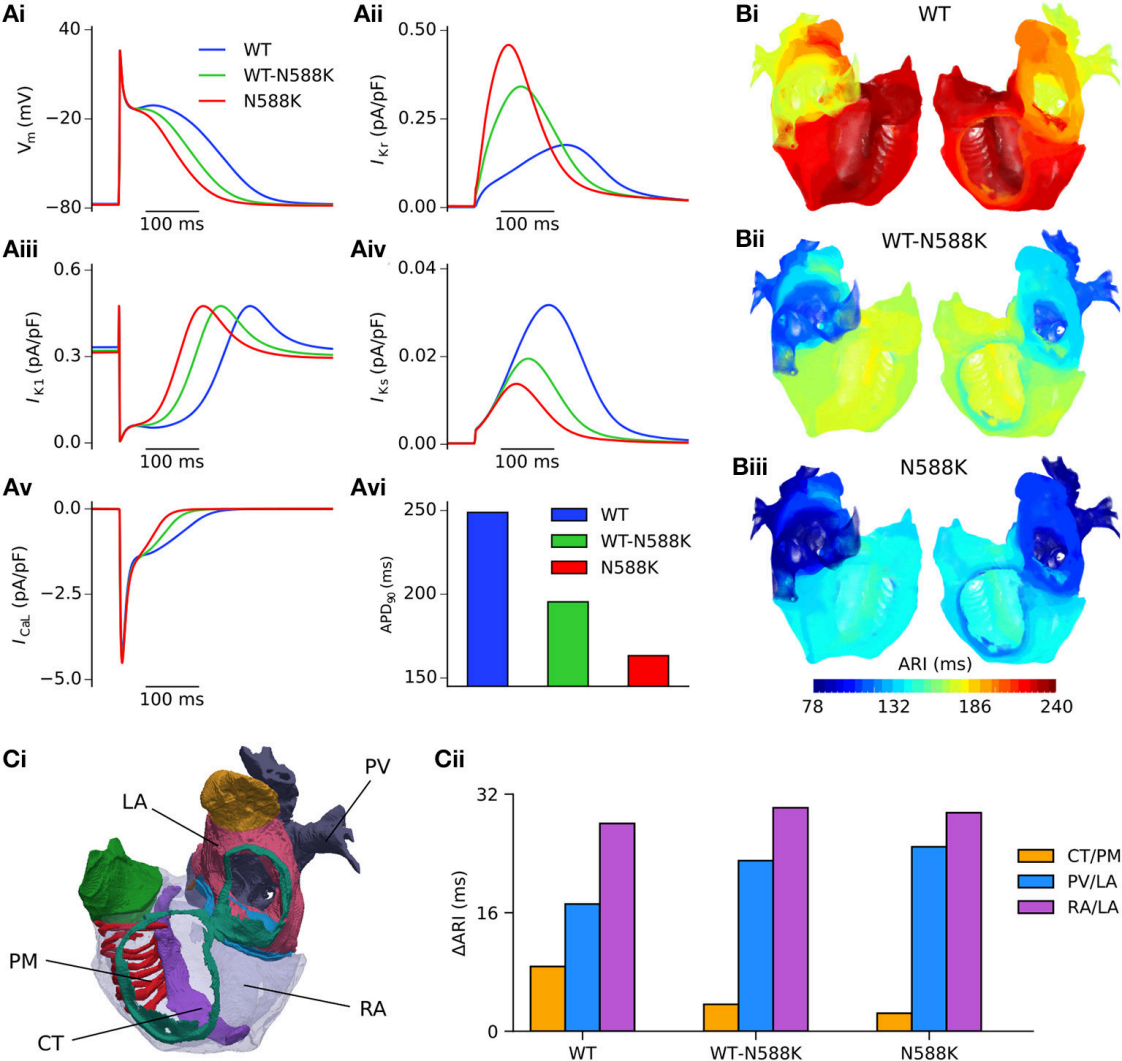
Effects of the N588K-hERG mutation at the single cell and whole human atria level. **(Ai)** Single cell action potential profiles under WT (blue), WT-N588K (green), and N588K (red) conditions at a pacing frequency of 1 Hz, with corresponding current traces for **(Aii)** *I*_Kr_, **(Aiii)** *I*_K1_, **(Aiv)** *I*_Ks_, and **(Av)** *I*_CaL_, and **(Avi)** a summary of the measured APD_90_. **(B)** Activation-recovery interval (ARI) maps under (i) WT, (ii) WT-N588K, and (iii) N588K conditions. **(Ci)** View looking into the cavities of the 3D anatomical human atria model, with the following regions highlighted: crista terminalis (CT), left atrium (LA), pectinate muscles (PM), pulmonary veins (PV), and right atrium (RA). **(Cii)** Measured dispersion of activation-recovery interval, ΔARI, between different atrial regions under WT and SQT1 mutation conditions.

Both SQT1 mutant conditions shortened the human atrial APD at a pacing rate of 1 Hz (Figure 3Ai), from 248.8 ms in the WT condition to 195.4 and 163.3 ms in heterozygous (WT-N588K) and homozygous (N588K) mutation conditions, respectively. This was due to loss of inactivation associated with the N588K-hERG mutation, which increased *I*_Kr_ early during the AP and served to accelerate the repolarisation process (Figure 3Aii). Action potential abbreviation reduced considerably the duration of the plateau phase, causing *I*_K1_ to contribute to terminal repolarisation earlier during the AP (Figure 3Aiii), and lesser activation and reduced contribution of *I*_Ks_ (Figure 3Aiv). The SQT1 mutation also decreased net *I*_CaL_ due to the abbreviated plateau phase (Figure 3Av), shortening the APD_90_ under heterozygous and homozygous conditions (Figure 3Avi). In tissue, accelerated repolarisation under SQT1 conditions shortened the ERP and consequently the excitation WL. At the organ scale (in the 3D anatomical human atria model), both heterozygous and homozygous forms of the N588K-hERG mutation decreased global ARI (Figure 3B), whilst preserving the global dispersion of ARI. Furthermore, whereas ΔARI was decreased between CT/PM regions, it was increased between PV/LA and RA/LA regions under SQT1 conditions (Figure 3C). A summary of the effects of the N588K-hERG mutation on multi-scale human atrial AP biomarkers is given in Table 1.

**Table 1:** A summary of multi-scale simulation results.

	**Single cell**		**1D**		**3D**	
	**APD_**90**_ (ms)**	**APD_**50**_ (ms)**	**ERP (ms)**	**WL (ms)**	**ΔARI (ms)**	**DF (Hz)**
**WT**	248.8	144.6	273	198.9	77	4.19*
**WT-N588K**	195.4	100.3	218	158.4	77	4.99
**N588K**	163.3	75.3	187	135.7	71	5.59

Analysis biomarkers at the single cell, 1D, and 3D levels under WT, WT-N588K, and N588K conditions at a pacing rate of 1 Hz. ΔARI refers to global dispersion of ARI. DF was measured from the first 5.0 s of activity. APD_90_ and APD_50_, Action Potential Duration at 90% and 50% repolarisation respectively; ERP, Effective Refractory Period; WL, Excitation Wavelength; ARI, Activation-Recovery Interval; DF, Dominant Frequency. The DF is an average computed from two simulations with clockwise and anti-clockwise initial conditions, except in the case marked with ^*^, in which only one value was used.

### Scroll Wave Dynamics in 3D Anatomical Human Atria Geometry

**Figure 4:**
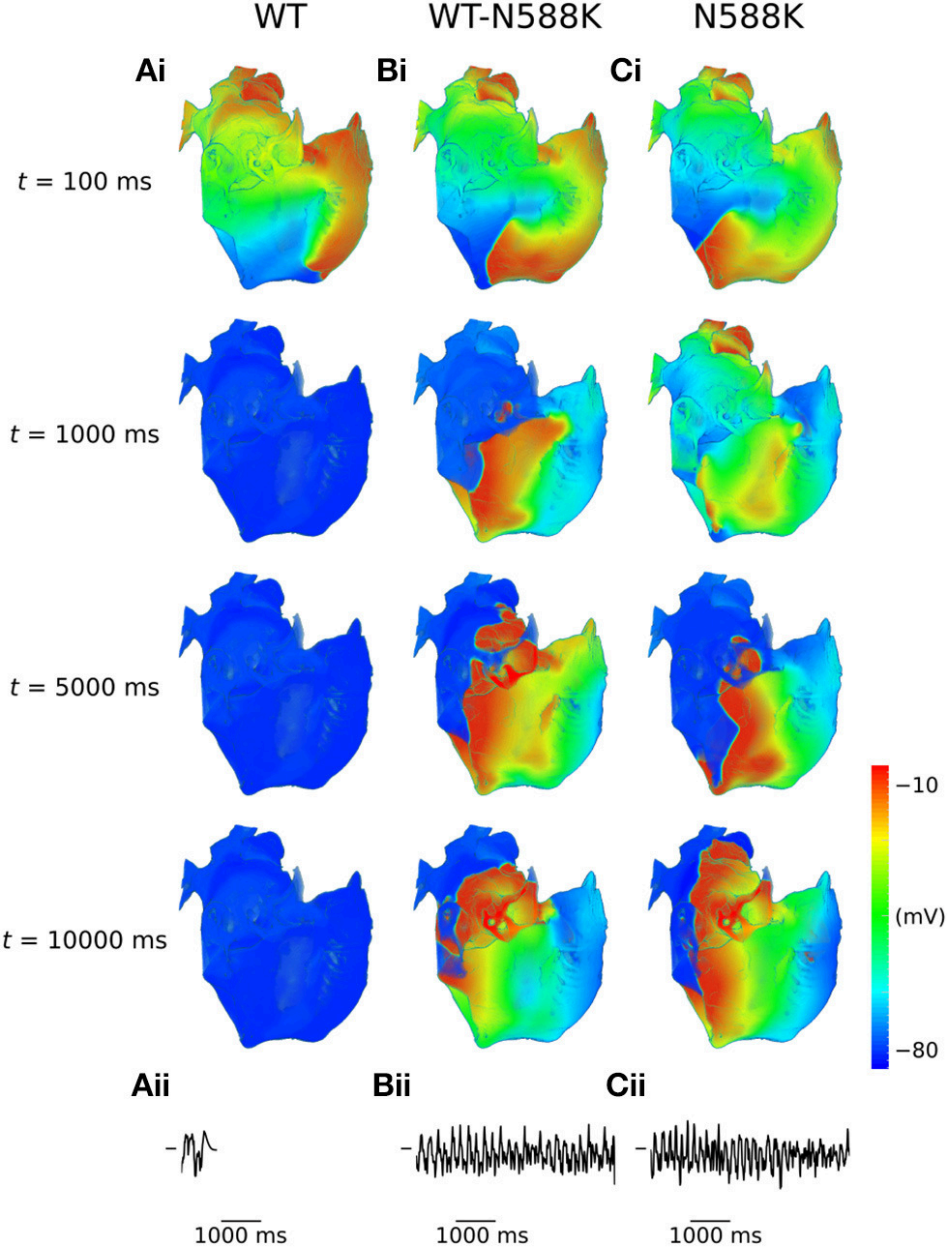
Scroll waves under WT and SQT1 mutation conditions in the 3D anatomical human atria model. (i) Evolution of scroll waves following initiation of re-entry in a clockwise configuration (from a RA posterior wall aspect) at times *t* = 100 ms, *t* = 1,000 ms, *t* = 5,000 ms, and *t* = 10,000 ms under **(A)** WT, **(B)** WT-N588K, and **(C)** N588K conditions, with (ii) corresponding pseudo ECGs taken from the first 5.0 s of re-entry simulations.

A summary of 3D scroll wave simulations in the anatomical human atria model under WT and SQT1 mutation conditions (in a clockwise configuration from a RA posterior wall aspect) is given in Figure 4. In the WT condition, scroll waves followed 2 transient, fragmented circuits around the RA, before self-terminating at ~0.7 s (Video S1), which precluded accurate measurement of the DF. In the WT-N588K condition, re-entrant wave activity sustained for the entire 10.0 s with a DF of 4.99 Hz, and was driven mostly by a macro re-entrant circuit around the right AVR, whilst also showing CT/isthmus driven activity in the RA (Video S2). Under homozygous N588K mutation conditions, scroll wave activity was also driven predominantly by a re-entrant circuit around the AVR, with occasional existence of multiple waves on the RA free wall (Video S3), and a DF of 5.59 Hz. A summary of simulations using anti-clockwise scroll wave initial conditions is shown in Figure S1, and an average of the DF from both simulations (where applicable) measured from the first 5.0 s of activity is given in Table 1.

### Effects of Class I Drugs on Human Atrial Action Potentials

**Figure 5:**
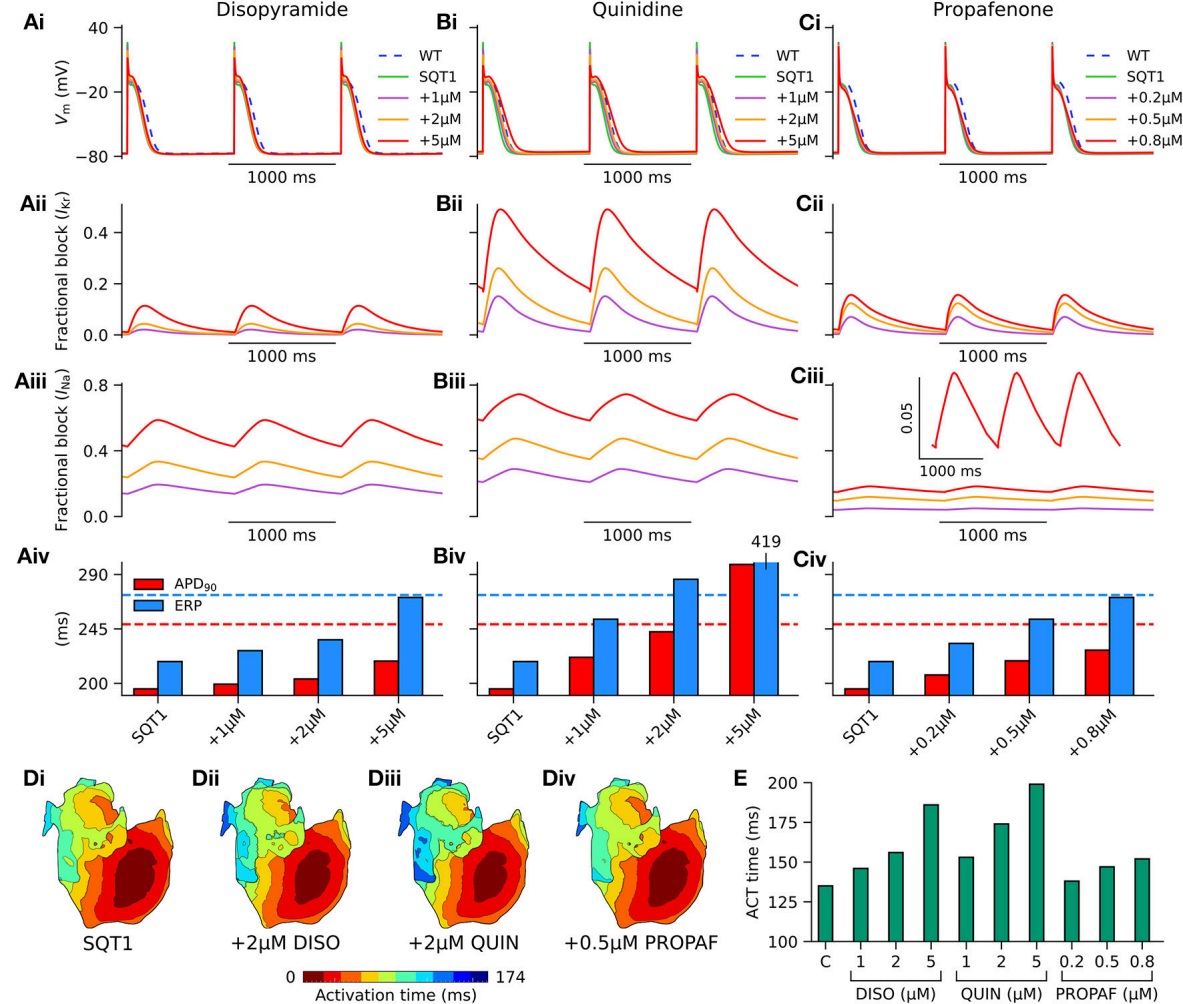
Effects of class I drugs on human atrial electrophysiology in SQT1. Effects of **(A)** disopyramide (DISO), **(B)** quinidine (QUIN), and **(C)** propafenone (PROPAF) on (i) single human atrial cell action potentials (APs) under wild type (WT) conditions (blue, dashed line), drug-free WT-N588K (SQT1) conditions (green, solid line), and SQT1 + varying drug concentrations (purple, orange, and red solid lines). Corresponding fractional block of (ii) *I*_Kr_ and (iii) *I*_Na_, and (iv) increase in APD_90_ (red) and effective refractory period (ERP; blue). **(D)** Activation sequences in the 3D anatomical human atria model under (i) drug-free SQT1 (control – C), (ii) SQT1 + 2 μM DISO, (iii) SQT1 + 2 μM QUIN, and (iv) SQT1 + 0.5 μM PROPAF conditions, with corresponding **(E)** activation (ACT) time summarized as a barchart. All simulations performed at a basic cycle length of 1,000 ms.

In order to assess the actions of class I anti-arrhythmic drugs on the human atria at the cellular level, the effects of applying various concentrations of disopyramide, quinidine, and propafenone were investigated on the single cell AP under heterozygous WT-N588K (hereinafter referred to simply as SQT1) conditions. Figure 5 shows AP profiles under drug-free SQT1 conditions and upon application of different concentrations of each drug at a pacing frequency of 1 Hz, with corresponding fractional block of *I*_Kr_ and *I*_Na_. Application of all concentrations of disopyramide and propafenone produced only modest prolongation of the APD, failing to restore it to that of the WT condition. Quinidine, on the other hand, was more effective at prolonging the APD due to extensive *I*_Kr_ block, with the highest concentration (5 μM) prolonging the APD beyond that of the WT level. In contrast, all three agents prolonged the ERP effectively, restoring it to (or exceeding) that of the WT level at the highest respective concentrations tested, due to additional ERP-prolonging effects of *I*_Na_ block.

Conduction slowing due to sodium channel block by the three drugs caused an increase in activation time in the 3D anatomical human atria model (Figure 5D), which was most prominent for quinidine and least prominent for propafenone at the concentrations tested. A summary of the effects of pharmacological modulation across multiple scales is given in Table 2. In addition, in the 1D tissue model, the rate-dependent effects of disopyramide, quinidine, and propafenone on the ERP, CV, and WL are shown in Figure S2. Quinidine was shown to produce the most potent effects on CV and ERP at all pacing rates. Propafenone exerted the weakest effects on ERP at fast pacing rates, but had the greatest propensity to promote beat-to-beat alternans.

**Table 2:** A summary of multi-scale pharmacological simulation results.

		**Single cell**	**1D**			**3D**
		**APD_**90**_ (ms)**	**ERP (ms)**	**CV (cm/s)**	**WL (mm)**	**ACT time (ms)**
**Drug-free**	**SQT1**	195.4	218	72.7	158.4	135
**DISO (μM)**	**1**	199.3	227	68.7	155.9	146
	**2**	203.6	236	65.4	154.3	156
	**5**	218.4	271	57.8	156.5	186
**QUIN (μM)**	**1**	221.5	253	66.6	168.4	153
	**2**	242.6	286	61.6	176.1	174
	**5**	298.3	419	49.4	207.2	199
**PROPAF (μM)**	**0.2**	206.9	233	72.0	167.8	138
	**0.5**	218.6	253	70.6	178.7	147
	**0.8**	227.4	271	69.1	187.4	152

*Analysis biomarkers at the single cell, 1D, and 3D levels at a pacing rate of 1 Hz under drug-free SQT1 conditions, and upon application of various concentrations of disopyramide (DISO), quinidine (QUIN), and propafenone (PROPAF). APD_90_, Action Potential Duration at 90% repolarisation; ERP, Effective Refractory Period; CV, Conduction Velocity; ACT time, Activation Time*.

### Pharmacological Effects of Class I Drugs on Re-entry Dynamics

**Figure 6:**
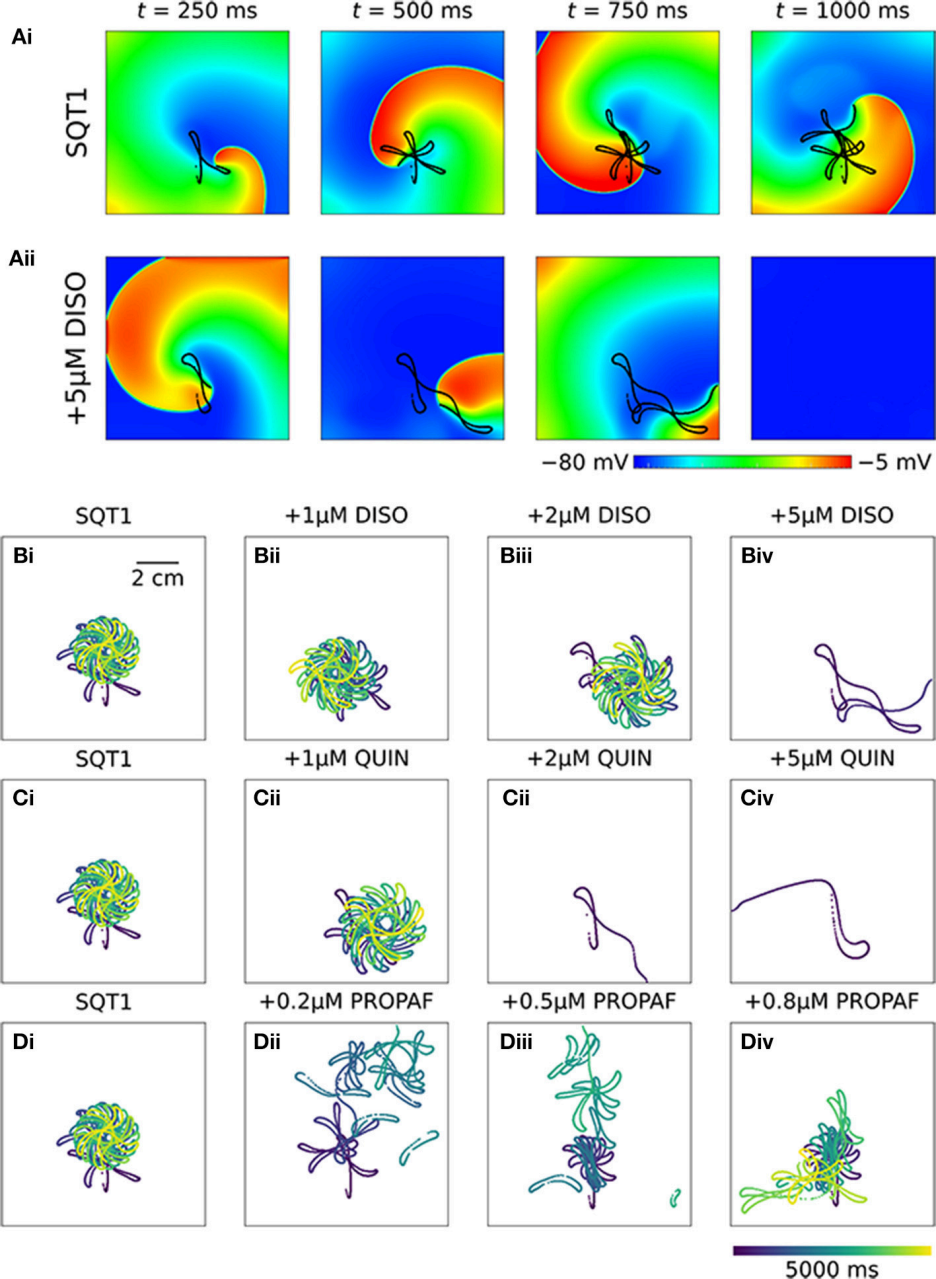
Re-entry dynamics in homogeneous 2D sheets upon application of disopyramide, propafenone, and quinidine in SQT1 mutation conditions. **(A)** Representative snapshots of re-entrant spiral waves under (i) drug-free SQT1 and (ii) SQT1 + 5 μM disopyramide (DISO) conditions at times, *t* = 250, 500, 750, and 1,000 ms following re-entry initiation, with spiral wave core trajectories superimposed onto membrane potentials in black. Spiral wave core trajectories over a 5,000 ms period under **(Bi, Ci, Di)** drug-free SQT1 conditions, and upon application of **(Bii)** 1 μM, **(Biii)** 2 μM, and **(Biv)** 5 μM DISO, **(Cii)** 1 μM, **(Ciii)** 2 μM, and **(Civ)** 5 μM quinidine (QUIN), and **(Dii)** 0.2 μM, **(Diii)** 0.5 μM, and **(Div)** 0.8 μM propafenone (PROPAF).

In 2D simulations, under drug-free WT conditions the initiated spiral wave failed to re-enter the tissue (not shown). Under drug-free SQT1 conditions, the initiated spiral wave sustained for the 5.0 s duration of the simulation, eventually settling into a stationary, epicycloidal trajectory (Figure 6Ai). Application of 5 μM disopyramide was sufficient to terminate re-entry, as the re-entry wavelength was increased to such an extent that the spiral wave meandered out of the tissue (Figure 6Aii). On the other hand, both 1 and 2 μM disopyramide did not terminate re-entrant activity, but did increase the area over which spiral waves meandered (from 0.35 mm^2^ ms^−1^ in the WT condition to 0.40 and 0.47 mm^2^ ms^−1^, respectively '(Figure 6B). Similarly, 1 μM quinidine increased the area of meander to 0.44 mm^2^ ms^−1^ without terminating re-entry, whereas 2 and 5 μM quinidine terminated re-entry without inducing wave break (Figure 6C). Application of 0.2 and 0.5 μM propafenone resulted in termination of re-entry by decreasing both the stationarity and stability of spiral waves (Figure 6D), whereas 0.8 μM propafenone did not terminate re-entry but did destabilize the re-entrant circuit and substantially increase the area of meander (to 0.75 mm^2^ ms^−1^).

**Figure 7:**
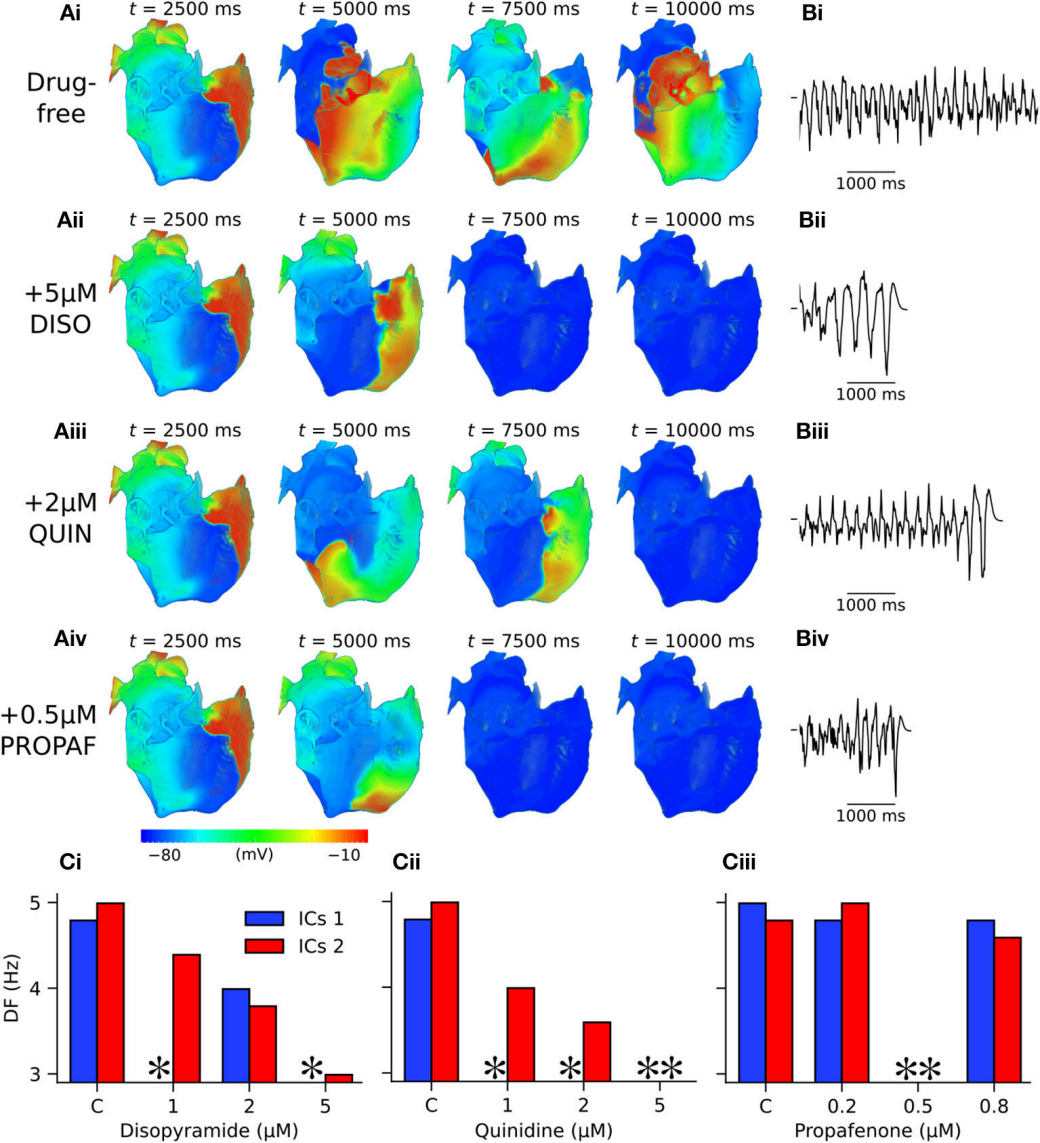
Arrhythmia termination in 3D anatomical human atria by representative concentrations of disopyramide, propafenone, and quinidine under SQT1 mutation conditions. **(A)** Snapshots of re-entrant scroll waves in a clockwise configuration (viewed from RA posterior wall) at various time points after initiation of a scroll wave in the 3D anatomical human atrial model under (i) drug-free WT-N588K conditions, and upon application of (ii) 5 μM disopyramide (DISO), (iii) 0.5 μM propafenone (PROPAF), and (iv) 2 μM quinidine (QUIN) conditions, with **(B)** corresponding pseudo ECG recorded during the final 5.0 s. In pharmacological simulation drugs were applied at *t* = 2,500 ms. **(C)** Barcharts summarizing DF upon application of various concentrations of (i) DISO, (ii) PROPAF, and (iii) QUIN for clockwise and anti-clockwise initial conditions (ICs 1 and ICs 2, respectively). ^*^ denotes simulations in which DF was not calculated due to occurrence of re-entry termination.

In 3D simulations using clockwise scroll wave initial conditions, quinidine terminated re-entry at all concentrations, and disopyramide terminated re-entry at concentrations of 1 and 5 μM (arrhythmia termination by 5 μM disopyramide and 2 μM quinidine are shown in Videos S4, S5, respectively). At a concentration of 2 μM disopyramide, the DF was reduced from 4.79 to 3.99 Hz (measured from the final 5.0 s of activity). Propafenone, on the other hand, was comparatively ineffective at reducing the DF at the concentrations tested, but terminated re-entry at a concentration of 0.5 μM (Video S6). Representative examples of arrhythmia termination by disopyramide, propafenone, and quinidine are shown in Figure 7. Disopyramide and quinidine produced a dose-dependent decrease in the DF which was greater than for propafenone (Figure 7C). In an anti-clockwise scroll wave configuration, the efficacy of anti-arrhythmic drugs was less favorable, with pharmacological conversion of re-entrant waves occurring only for 0.5 μM propafenone and 5 μM quinidine. A quantitative summary of all 2D and 3D re-entry simulations is given in Table S5.

## Discussion

In this study, pathophysiological effects of the SQT1-related N588K-hERG mutation on human atrial electrophysiology were assessed, investigating mechanisms of increased susceptibility to the development of atrial arrhythmias in multi-scale cellular and tissue models incorporating electrical and anatomical heterogeneities. Furthermore, the actions of the class I anti-arrhythmic drugs disopyramide, quinidine, and propafenone were assessed in the context of sustained atrial arrhythmic excitations mediated by the SQT1 N588K mutation using drug simulations including binding kinetics, altered potency of *I*_Kr_/hERG block in SQT1, and multiple ion channel block. Both quinidine and disopyramide have demonstrated efficacy in QT interval prolongation in the setting of SQT1 (Gaita et al., 2004; Schimpf et al., 2007; Mizobuchi et al., 2008; Giustetto et al., 2011; Hu et al., 2017), whereas propafenone has been used in 3 patients for management of episodes of paroxysmal AF mediated by SQT1, which prevented recurrence of arrhythmias but did not prolong the QT interval (Hong et al., 2005; Bjerregaard et al., 2006). This study provides clinically-relevant insights into pharmacology of SQT1 by evaluating and comparing the actions of all three drugs in the context of accelerated atrial repolarisation and arrhythmias mediated by SQT1, offering an important step toward *in silico* optimisation of pharmacological therapy in this context.

### Main Findings

**Figure 8:**
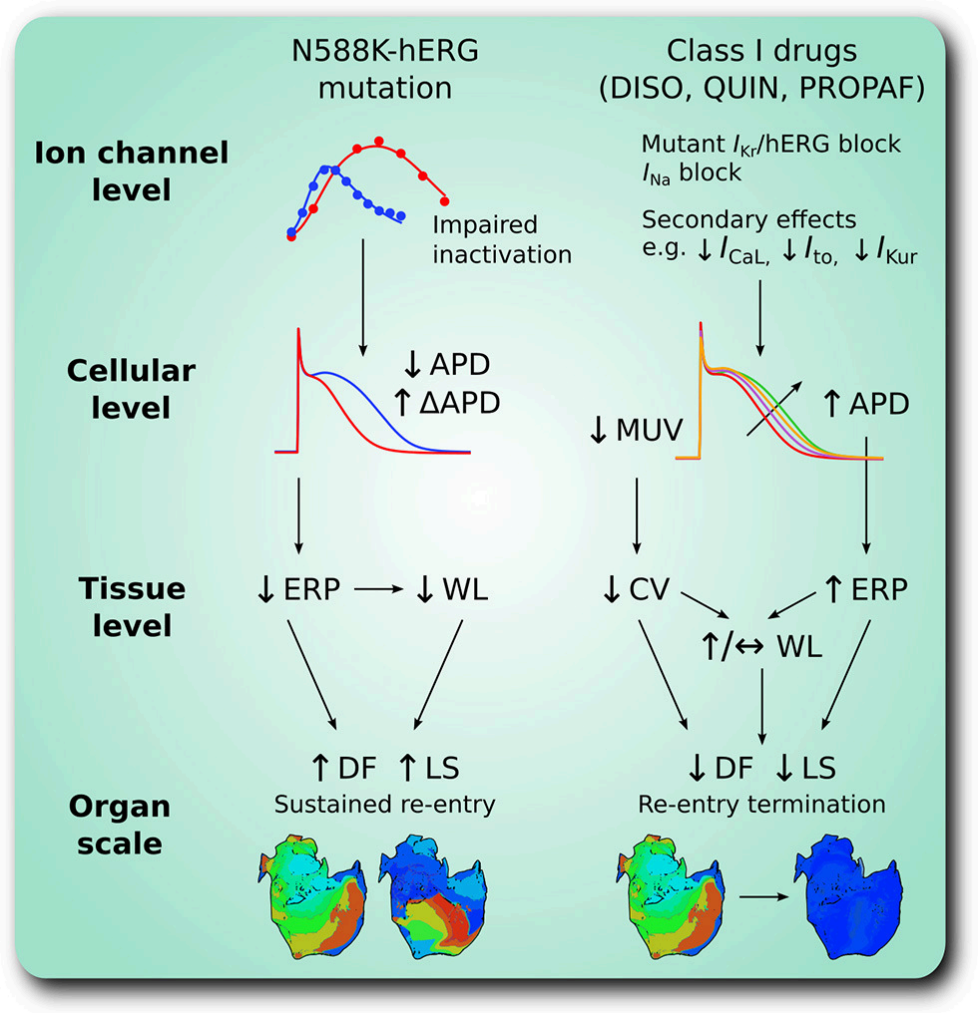
A schematic summary of pro-arrhythmic effects of the N588K-hERG mutation in human atria and anti-arrhythmic effects of class I drugs. At the ion channel level, the N588K mutation impairs hERG channel inactivation, increasing *I*_Kr_ which shortens the action potential duration (APD) at the cellular level, and increases its dispersion, ΔAPD, between certain regions of the human atria. In tissue, shortened APD corresponds to shortened effective refractory period (ERP) and excitation wavelength (WL), promoting sustenance of arrhythmias at the organ scale through higher dominant frequency (DF) and lifespan (LS) of re-entry. Class I drugs disopyramide (DISO), quinidine (QUIN), and propafenone (PROPAF) exert effects at the ion channel level by blocking *I*_Kr_ and *I*_Na_, in addition to secondary actions. This prolongs APD and ERP, and reduces maximum upstroke velocity (MUV) and conduction velocity (CV) at the cellular and tissue levels, respectively. At the organ scale these pharmacological actions promote arrhythmia rate and rhythm control through reduced DF and LS of re-entry.

The major findings presented in this study are as follows. (1) Heterozygous and homozygous forms of the SQT1-linked N588K-hERG mutation shortened significantly the APD of human atrial cells, whilst preserving global dispersion of ARI, and increasing ΔARI between certain regions of the human atria. (2) The dominant frequency and lifespan of re-entry in 3D arrhythmia simulations was increased by heterozygous and homozygous forms of the N588K mutation. (3) Disopyramide, quinidine, and propafenone all produced ERP prolongation in the setting of SQT1, the extent of which was greatest for quinidine. (4) In 2D simulations, disopyramide and quinidine terminated re-entry at high concentrations due to increased re-entry wavelength, whereas propafenone terminated re-entry in a non-dose-dependent manner, by inducing secondary waves. (5) In 3D simulations, the DF of re-entry was reduced in a dose-dependent manner for clinically-relevant doses of disopyramide and quinidine, and propafenone to a lesser extent. (6) All three anti-arrhythmic agents demonstrated some efficacy in pharmacological rhythm control, most frequently terminating re-entry in the order quinidine > propafenone = disopyramide. A summary of findings regarding pro-arrhythmic mechanisms of the N588K-hERG mutation in human atria and anti-arrhythmic actions of selected class I drugs is given in Figure 8.

### SQT1 Mutant *I*_Kr_ Promotes Human Atrial Arrhythmogenesis

The fact that the N588K-hERG mutation is associated with both a short QT interval and reports of AF (Hong et al., 2005) suggests that pathophysiological mechanisms leading to QT interval shortening in SQT1 also affect the atria and may promote AF. In an experimental model of SQT1, application of 20 μM of the *I*_Kr_ agonist PD-118057 shortened the APD in both CT and PM cell types of canine atrial tissue (Nof et al., 2010), as well as increasing APD dispersion between them. In the present study, at the cellular level heterozygous and homozygous forms of the N588K-hERG mutation produced a shortening of the human right atrial APD of 53.4 and 85.5 ms, respectively–values which are consistent with a previous simulation study (Loewe et al., 2014b). At the tissue level, global ΔARI was unaffected by the WT-N588K mutation condition, and was slightly decreased by the homozygous form. The model predicted an increase in ΔARI between PV/LA and RA/LA regions for both heterozygous and homozygous forms of the N588K mutation (Figure 3C), but not between CT/PM regions. This may be due to the fact that the effects of the hERG activator PD-118057 and of the N588K mutation on *I*_Kr_/hERG are different: the former increases *I*_Kr_/hERG channel open probability without altering gating kinetics (Zhou et al., 2005), whereas the latter alters kinetics through profound attenuation of inactivation (Cordeiro et al., 2005; McPate et al., 2005).

Interestingly, there is clinical evidence of a LA-RA gradient in the DF in paroxysmal AF (Lazar et al., 2004), which may be underlain by APD differences between the RA and LA in pathological conditions which promote AF (Voigt et al., 2010). Furthermore, regional differences in APD between PV/LA regions have been identified previously as high frequency excitation or microreentrant sources underlying AF (Mandapati et al., 2000; Arora et al., 2003; Kumagai et al., 2004)–this suggests that localized increases in the spatial dispersion of repolarisation could be a mechanism by which atrial arrhythmogenesis is promoted in SQT1, as suggested in the previous experimental study (Nof et al., 2010).

In a previous simulation study (Loewe et al., 2014b), the N588K-hERG mutation in a 1:1 mutant to WT ratio was shown to increase inducibility and lifespan of rotors in idealized 2D sheets of human atrial tissue, but did not permit sustained re-entry. By contrast, in the present study, the heterozygous WT-N588K (SQT1) condition permitted the sustenance of spiral waves in 2D simulations. This may be due to several differences in the modeling, one of which is that the CRN model used in that study (Loewe et al., 2014b) produces an ERP restitution curve which is around 50 ms higher than available experimental data (Wilhelms et al., 2013), making re-entry more difficult to sustain, whereas the CNZ model ERP restitution curve lies mostly within the experimental range (Figure S3). In this study, the presence of realistic structure and heterogeneous electrophysiology, in addition to the cellular electrophysiological substrate induced by the N588K-hERG mutation, also favored the sustenance of re-entrant scroll waves in the 3D anatomical human atria model (using both clockwise and anti-clockwise scroll wave initial conditions), and could both prolong or shorten the lifespan of re-entrant excitations under pharmacological modulation conditions when compared directly to observations in 2D homogeneous sheets. This highlights the value of including both approaches in probing the arrhythmia substrate associated with genetic mutations in human atria, as behavior in heterogeneous 3D anatomical models cannot necessarily be predicted from homogeneous 2D simulations, and vice versa. It should be noted nonetheless that electrical activity observed in 3D re-entry simulations in this study remained relatively organized and generally not driven by multiple wavelets. This lack of complex, chaotic behavior which is observed in persistent AF is consistent with reports of paroxysmal AF in some patients with the N588K-hERG mutation (Hong et al., 2005) – addition of electrical and intracellular gap junction remodeling or fibrosis in patients may be required to promote degeneration of paroxysmal AF into persistent AF.

### Class I Drugs May Represent Useful SQT1-Specific Pharmacotherapy for AF

Despite the prevalence of AF and decades of research, anti-arrhythmic therapies for AF continue to have limited efficacy and safety (Workman et al., 2011). In this study, the class I anti-arrhythmic drugs disopyramide, quinidine, and propafenone were shown to be only moderately effective in the management of atrial arrhythmias mediated by the SQT1-linked N588K-hERG mutation, although it should be noted that the 7.5 s duration of 3D drug simulations is much shorter than clinical time scales. Pharmacological rhythm control occurred under application of both disopyramide and quinidine, but in an unpredictable, non-dose-dependent way, as observed in our previous *in silico* study (Whittaker et al., 2017a). One mechanism of re-entry termination involving reduction of the excitable gap under application of 5 μM quinidine is shown in Figure S4.

Both disopyramide and quinidine reduced consistently the DF of re-entry in a dose-dependent manner, although as in (Whittaker et al., 2017a) quinidine was more effective than disopyramide at rate control in the setting of SQT1, likely due to its more potent block of *I*_Kr_ (McPate et al., 2008) and *I*_Na_ (Koumi et al., 1992). Propafenone was comparatively ineffective at controlling the rate of atrial arrhythmias under the WT-N588K mutation condition, although did demonstrate some efficacy in re-entry termination. Figure S5 shows a mechanism of pharmacological rhythm control by 0.5 μM propafenone, in which secondary waves induced by propafenone extinguished all re-entrant pathways. This is consistent with the use of propafenone to manage paroxysmal AF in SQT1 patients (Hong et al., 2005; Bjerregaard et al., 2006), which maintained 3 patients free of arrhythmia recurrence during follow up. However, as propafenone has been reported to have no prolonging effect on the QT interval in the setting of SQT1 (Hong et al., 2005)— an effect which was demonstrated with variable success using two leading human ventricle models (ten Tusscher and Panfilov, 2006; O'Hara et al., 2011) in Figure S6—it is likely that quinidine and disopyramide will remain more suitable pharmacotherapies.

In this study, cellular and tissue level simulations (in a 1D model) alone did not serve as good predictors of the overall effects of anti-arrhythmic drugs in the electrically-heterogeneous, 3D anatomical whole atria model, consistent with previous modeling studies (Varela et al., 2016; Whittaker et al., 2017b). For example, disopyramide was shown not to strongly affect the excitation WL in RA tissue (Table 2; Figure S2), and yet it decreased reliably the DF of re-entrant excitations under heterozygous N588K mutation conditions in 3D simulations. This is likely due to the fact that scroll waves were largely anatomically driven by a typical macro re-entrant atrial flutter/tachycardia circuit (Lee et al., 2012) which was larger than the functional WL; effects of disopyramide on CV were thus more important. In contrast, propafenone produced only modest changes to the CV and was thus less effective at rate control in the model, but did terminate re-entry under conditions in which the re-entrant circuit was abolished by interaction with secondary waves (Figure S5).

### Insights Into the Efficacy of Class I Drugs in SQT1

Further support for quinidine as a useful pharmacotherapy for AF in the setting of SQT1 comes from the experimental use of the *I*_Kr_ agonist PD-118057 model (Nof et al., 2010). That study suggested that the combined blocking actions on *I*_Kr_ and *I*_Na_ are what made quinidine effective at preventing sustained AF in canine right atrial tissue preparations, as neither *I*_Kr_ nor *I*_Na_ block alone (by E-4031 or lidocaine, respectively) was effective at terminating AF. Similarly, in our previous simulation study of ventricular pharmacotherapy for SQT1 we showed that combined block of *I*_Na_ and *I*_Kr_ by disopyramide and quinidine prolonged the ERP to a greater extent than block of *I*_Na_ or *I*_Kr_ alone (Whittaker et al., 2017a). In this study, we sought to determine whether the greater ERP prolongation associated with the synergistic combination of *I*_Na_ and *I*_Kr_ block by class I drugs would also translate to greater efficacy in terminating re-entry in a 2D sheet of SQT1 mutant human atrial tissue. Figure S7 shows that the actions of 5 μM of a hypothetical drug with disopyramide kinetics for *I*_Na_ and *I*_Kr_ block were able to terminate re-entry, whereas *I*_Na_ or *I*_Kr_ block alone did not, as combined block produced the greatest prolongation of the ERP. The spiral wave trajectories highlight the fact that *I*_Na_ block in particular destabilized the re-entrant circuit, which, when combined with *I*_Kr_ block, caused the spiral wave to meander out of the tissue boundaries (Video S7). The findings from *in silico* and *in vitro* experiments thus suggest that the combination of prolonged APD and refractoriness arising from K^+^ and Na^+^ channel inhibition is likely to be valuable in the setting of SQT1. Though selective blockers of *I*_Ks_ and *I*_K1_ are not yet clinically-available, this combination may also warrant investigation as a pharmacotherapeutic strategy for other forms of K^+^-linked SQTS (SQT2 and SQT3).

## Limitations

There are a number of limitations of the simulations presented in this study. The 3D anatomical human atria model which was employed for simulations in this study incorporated rule-based fiber orientations (Krueger et al., 2011), which may not capture sufficiently the complex cardiac microstructure of the human atria (Stephenson et al., 2017) which could contribute to the arrhythmia substrate. Whilst several hERG mutations have now been identified in SQT1 (Hancox et al., 2018), the present study focused only on the N588K-hERG mutation (which produces a more marked effect on hERG kinetics than some other mutations, Hancox et al., 2018), so the results here may not apply to all forms of SQT1. However, recently the S631A-hERG mutation (previously engineered for hERG structure-function studies), which produces a similar extent of attenuation of inactivation to N588K-hERG (McPate et al., 2008) has been reported in an SQT1 family (Akdis et al., 2018), and thus the findings of this study are likely also to be relevant to that form of SQT1. An additional potential limitation arises from the fact that mechanical contraction was not considered, which has been reported previously to be impaired in the setting of SQT1 in single human atrial cell simulations (Whittaker et al., 2015) as well as in organ-scale ventricle simulations (Adeniran et al., 2013), and based on clinical measurements (Frea et al., 2015).

The models of disopyramide and quinidine used in this study were adopted from (Whittaker et al., 2017a), in which the effects of anti-arrhythmic drugs were assessed in the setting of SQT1 in the human ventricles. However, both agents also exhibit modest anti-cholinergic effects in the atria (Nakajima et al., 1989) which were not accounted for, due to the absence of a formulation for acetylcholine-activated potassium current, *I*_K, ACh_, in the CNZ model. Similarly, the β-adrenergic receptor blocking effects of propafenone were not considered (McLeod et al., 1984). Inclusion of these factors could give some insight into the response of the SQT1 phenotype to anti-arrhythmic drugs in the presence of autonomic modulation, and could give more favorable results in terms of arrhythmia prevention, especially for propafenone, which may be particularly effective against AF triggered by increased vagal tone. In addition, dynamic effects of agents due to the influence of circadian variations in drug concentration and heart rate on bioavailability were not investigated, which can give further insights into differing clinical efficacies of drugs (Loewe et al., 2014a). In relation to this, a further, more general limitation is that translation of drug concentrations from experiments and computer simulations to meaningful clinical concentrations is potentially problematic. Finally, whilst the 1:1 mutant to WT ratio, which was intended to represent the heterozygous state of the proband, may represent an oversimplification of the real hERG channel population in SQT1, the approach adopted in this and our previous studies (Adeniran et al., 2011, 2017; Whittaker et al., 2017a) reproduced quantitatively QT interval shortening and T wave morphology in SQTS conditions which was concordant with clinical observations.

## Conclusions

The simulations performed in this study further substantiate a causative link between the SQTS-related N588K mutation and APD/ERP shortening in human atria, as well as increased spatial dispersion of repolarisation, which promotes development of AF. In 3D human atria simulations which included heterogeneous anatomy and electrophysiology, the N588K mutation was shown to increase the dominant frequency and lifespan of re-entrant excitation beyond that observed in WT conditions. Pharmacological simulations demonstrated that disopyramide and quinidine were more effective at rate control than propafenone in the setting of SQT1, and quinidine was most effective at rhythm control. Combined block of *I*_Na_ and *I*_Kr_ by a hypothetical drug was shown to be more beneficial in terms of re-entry termination in a 2D sheet of human atrial tissue than *I*_Kr_ or *I*_Na_ block alone in this context, suggesting useful targets for future pharmacotherapies. The multi-scale integrative cardiac modeling approach adopted in this study serves as a useful paradigm for optimisation of pharmacological therapy, allowing investigation of how genetic defects at the ion channel level influence organ scale propagation, arrhythmogenesis, and response to pharmacotherapies.

## Author Contributions

DW, JH, and HZ conceived the experiments and wrote the manuscript. DW developed and validated computer models and performed numerical experiments and analysis.

### Conflict of Interest Statement

The authors declare that the research was conducted in the absence of any commercial or financial relationships that could be construed as a potential conflict of interest.
