# Supplementary material for: In silico Assessment of Pharmacotherapy for Human Atrial Patho-Electrophysiology Associated With hERG-Linked Short QT Syndrome
Source: Front Physiol. 2019 Jan 11;9:1888. doi: 10.3389/fphys.2018.01888 (PMC6336736; doi:10.3389/fphys.2018.01888)
Supplement: Supplementary file 8 [file Data_Sheet_1.pdf]

Supplementary Material

In silico assessment of pharmacotherapy for human atrial patho-electrophysiology associated with hERG-linked short QT syndrome

Dominic G. Whittaker, Jules C. Hancox*, Henggui Zhang*

*** Correspondence:** Corresponding Author(s):
jules.hancox@bristol.ac.uk (JH); henggui.zhang@manchester.ac.uk (HZ)

1. Supplementary Figures

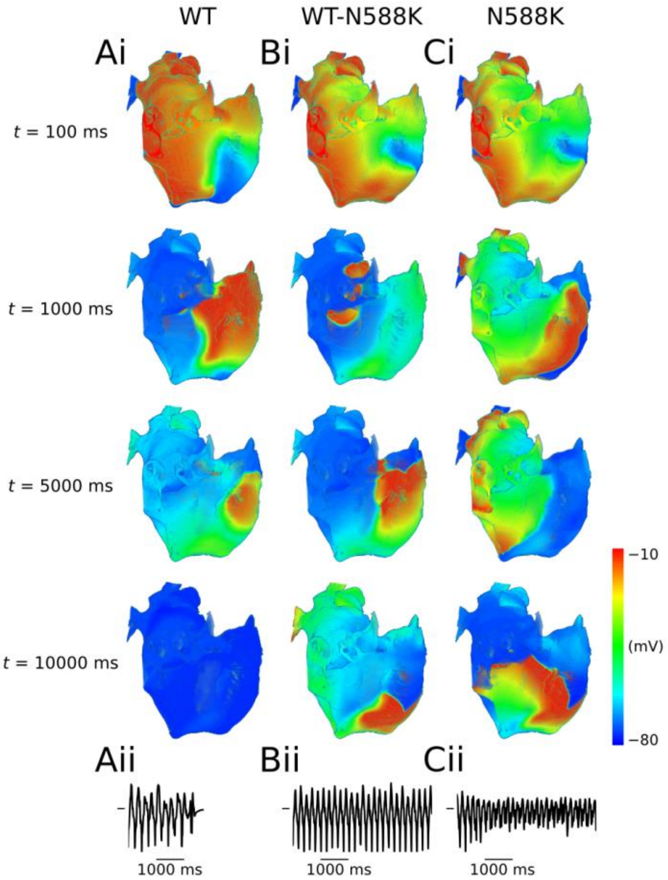

Figure S1: Scroll waves under WT and SQT1 mutation conditions in the 3D anatomical human atria model using alternative initial conditions. (i) Evolution of scroll waves following initiation of re-entry in an anti-clockwise configuration (from a RA posterior wall aspect) at times t = 100 ms, t = 1000 ms, t = 5000 ms, and t = 10000 ms under (A) WT, (B) WT-N588K, and (C) N588K conditions, with (ii) corresponding pseudo ECGs taken from the final 5.0 s of re-entry simulations.

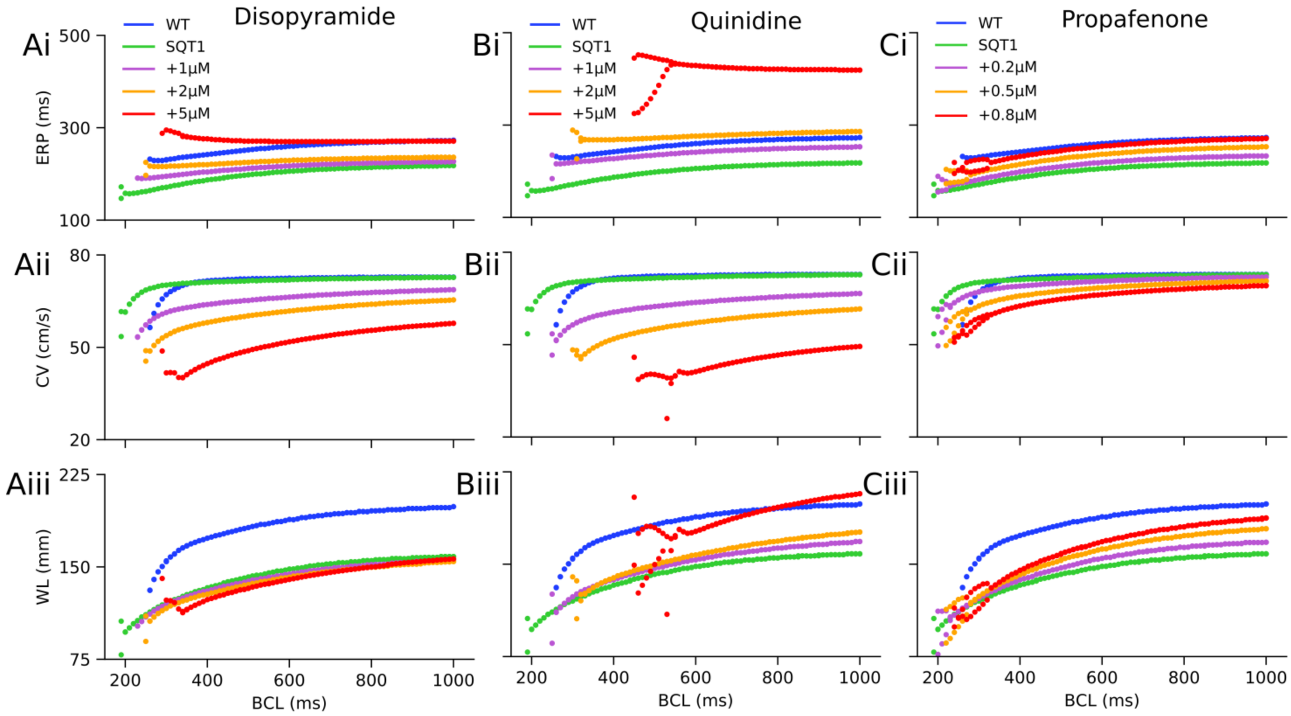

Figure S2: Rate-dependent effects of class I drugs on human atrial tissue. Effects of various concentrations (purple, orange, and red for increasing concentrations) of (A) disopyramide, (B) quinidine, and (C) propafenone on SQT1 (green) mutant atrial tissue, measured using a 1D strand model. The effects of drugs on the (i) effective refractory period (ERP), (ii) conduction velocity (CV), and excitation wavelength (WL) are shown, and compared with the wild type (WT; blue) for reference. For each basic cycle length (BCL), the two final action potentials were analysed to account for beat-to-beat alternans.

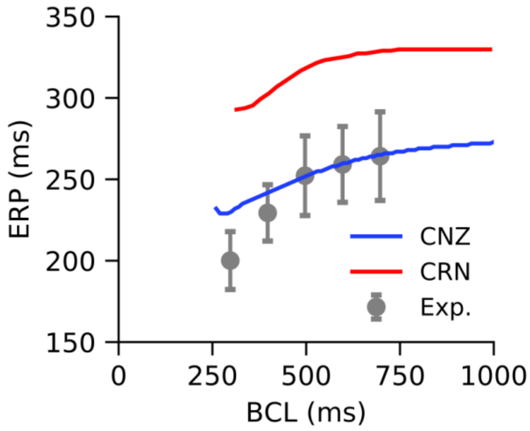

Figure S3: Comparison of human atrial model ERP restitution curves with experimental data. Effective refractory period (ERP) against basic cycle length (BCL) in the Colman-Ni-Zhang (CNZ) (Ni et al., 2017) and Courtemanche-Ramirez-Nattel (CRN) (Courtemanche et al., 1998) models of the human atrial action potential, compared with human atrial experimental data (Yu et al., 1999).

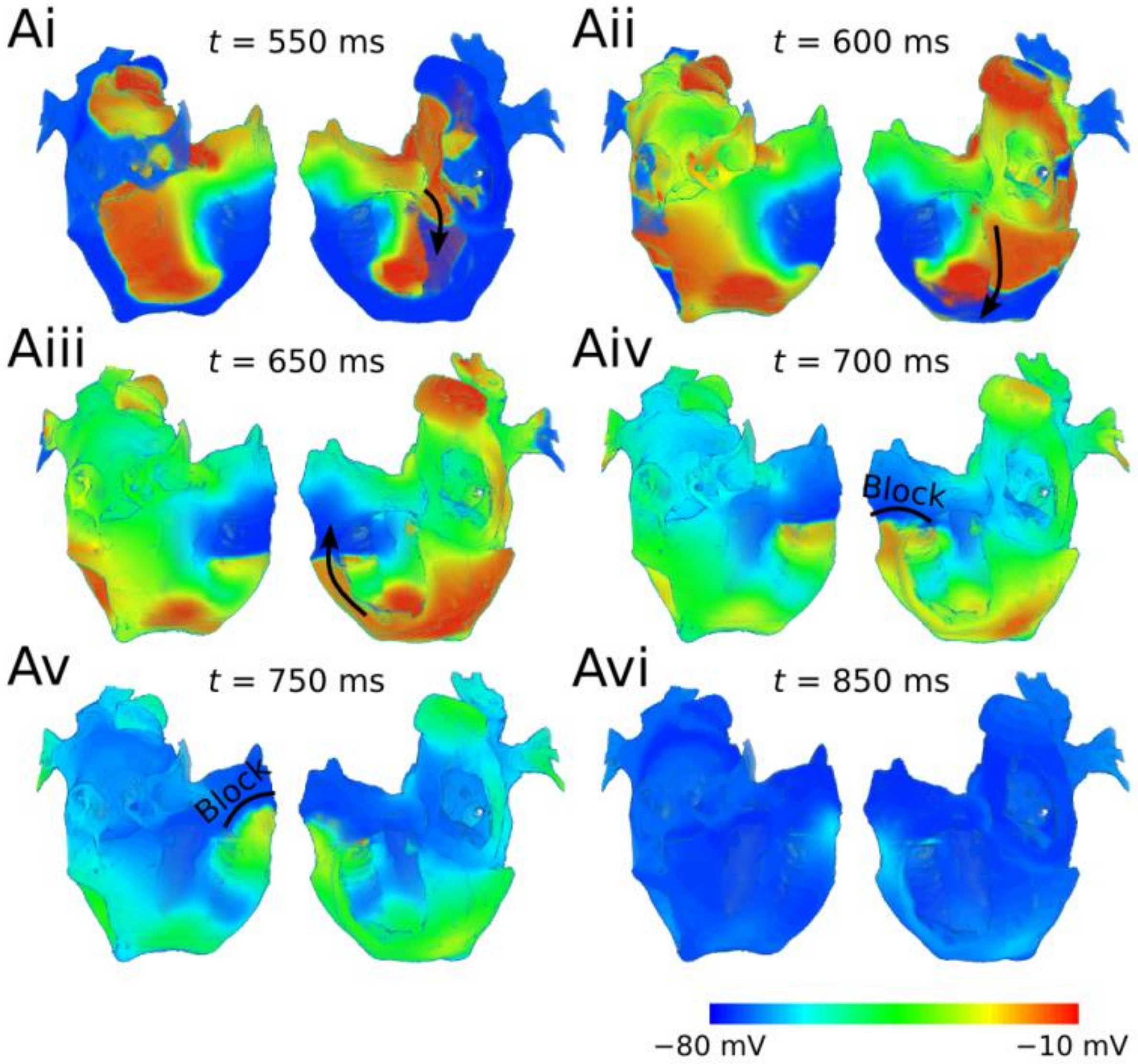

Figure S4: Mechanism of arrhythmia termination by quinidine in SQT1. A mechanism of arrhythmia termination by 5 μM quinidine in the setting of SQT1 is shown from two views – looking at the RA posterior wall and into the cavities. At times *t* = 550 ms (Ai), *t* = 600 ms (Aii), and *t* = 650 ms (Aiii) following application of quinidine, excitations followed a re-entrant circuit around the right atrio-ventricular annulus. At times *t* = 700 ms (Aiv) and *t* = 750 ms (Av) the excitation wave encountered refractory tissue and thus conduction blocks, ultimately leading to self-termination of re-entry by *t* = 850 ms (Avi). Black arrows denote the direction of propagation of selected wavefronts.

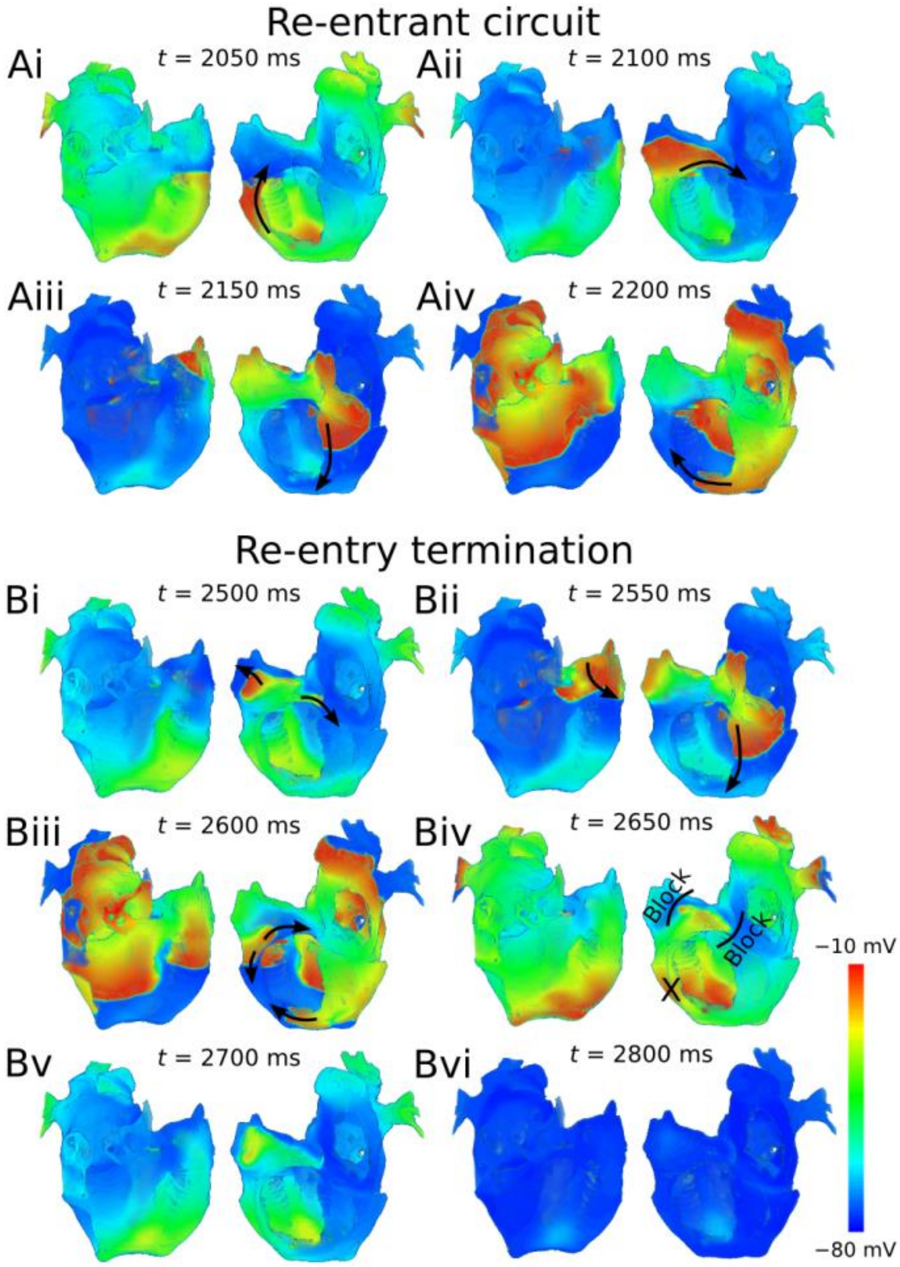

Figure S5: Mechanism of arrhythmia termination by propafenone in SQT1. A mechanism of arrhythmia termination by 0.5 μM propafenone in the setting of SQT1 is shown from two views – looking at the RA posterior wall and into the cavities. At times *t* = 2050 ms (Ai), *t* = 2100 ms (Aii), *t* = 2150 ms (Aiii), and *t* = 2100 ms (Aiv) following application of propafenone, excitations followed a re-entrant circuit around the right atrio-ventricular annulus. At times *t* = 2500 ms (Bi), *t* = 2550 ms (Bii), and *t* = 2600 ms (Biii) multiple waves destabilized the re-entrant circuit, leading to conduction blocks at *t* = 2650 ms (Biv) and *t* = 2700 ms (Bv), and ultimately self-termination of re-entry at *t* = 2800 ms (Bvi). Black arrows denote the direction of propagation of selected wavefronts, and X denotes collision of waves.

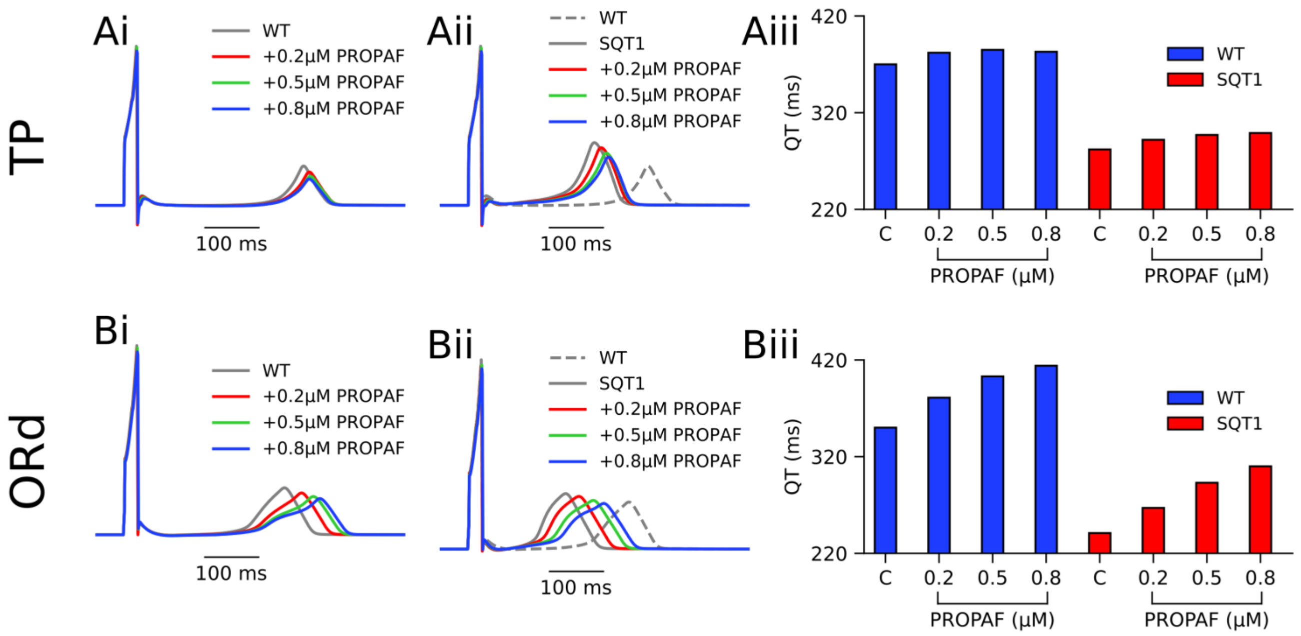

Figure S6: Effects of propafenone on leading human ventricular cell models. The effects of 0.2 μM (red), 0.5 μM (green), and 0.8 μM (blue) propafenone (PROPAF) on (i) wild type (WT) tissue and (ii) SQT1 mutant tissue are shown for the (A) Tusscher-Panfilov (TP) model (Tusscher and Panfilov, 2006) and (B) O'Hara-Rudy dynamic (ORd) model (O'Hara et al., 2011) of human ventricular cells. (iii) Bar charts showing corresponding changes in the QT interval compared to the respective control (C) – WT (blue) or SQT1 (red).

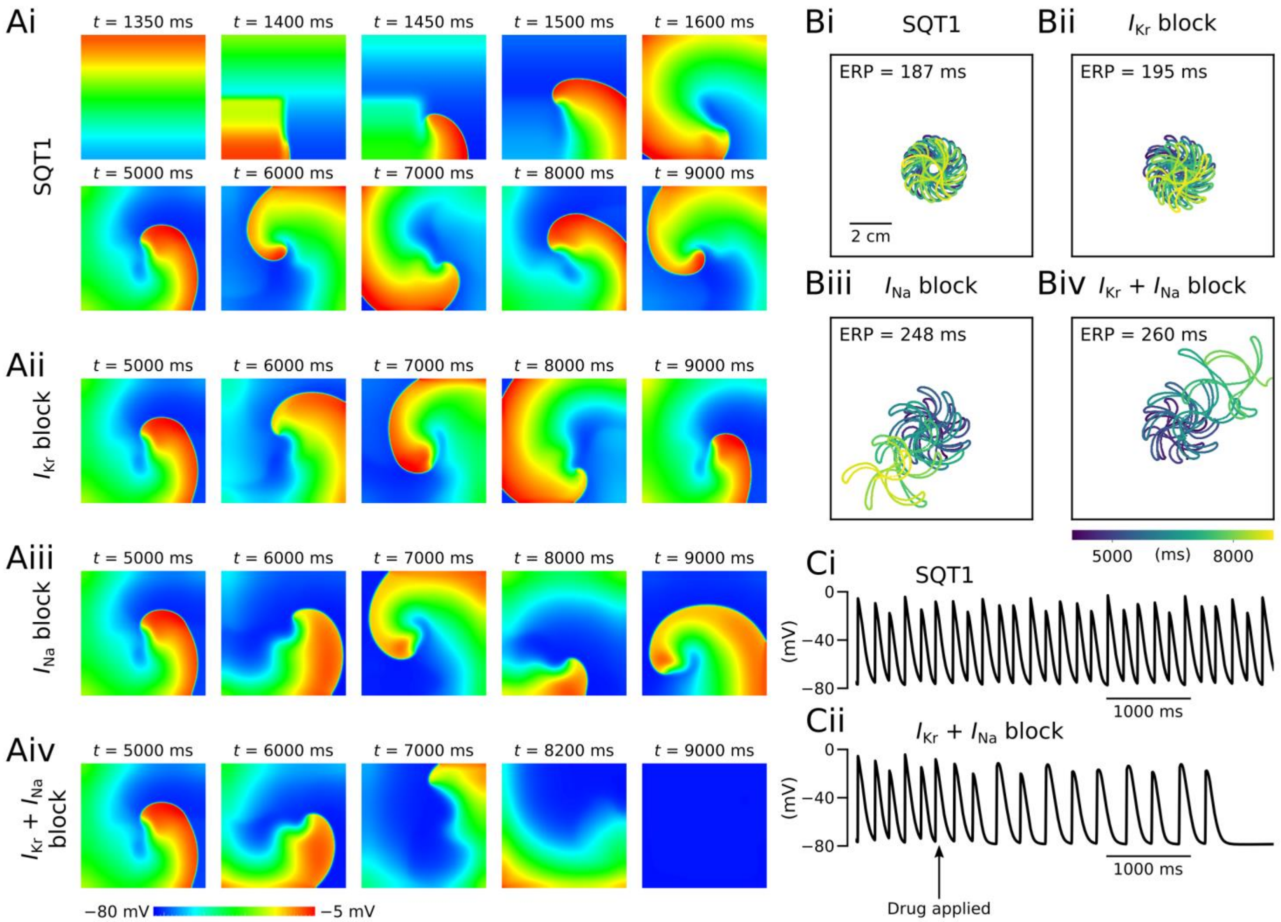

Figure S7: Re-entry termination by combined I_{Kr} and I_{Na} block under SQT1 conditions. (A) Snapshots of spiral waves in idealised 2D sheets of human atrial tissue in (i) drug-free SQT1 conditions, (ii) I_{Kr} block, (iii) I_{Na} block, and (iv) I_{Kr} + I_{Na} block conditions, and (B) corresponding spiral wave core trajectories, with effective refractory period (ERP) at BCL = 400 ms given. (C) Action potentials in (i) SQT1 and (ii) I_{Kr} + I_{Na} block conditions over a 5000 ms period from t = 4000 to 9000 ms. Re-entry was initiated using an S1-S2 protocol, and 5μM of hypothetical drugs with disopyramide I_{Kr}/I_{Na} block kinetics were applied at t = 5000 ms.

2. Supplementary Tables

Table S1. Ionic differences in the CNZ family of regional cell models.

	G_{CaL}	G_{to}	G_{Kur}	G_{Na}	G_{Kr}	G_{Ks}	G_{K1}	Source
**CT**	2.0	1.0	1.0	1.0	1.0	1.0	1.0	(Feng et al., 1998)
**BB**	2.2	1.0	1.0	1.0	1.0	1.0	1.0	(Burashnikov et al., 2004; Feng et al., 1998)
**PM**	0.94	1.0	1.0	1.0	1.0	1.0	1.0	(Feng et al., 1998)
**AVR**	0.67	0.6	1.0	1.0	1.63	1.0	1.0	(Feng et al., 1998)
**RAA**	1.0	0.68	1.0	1.0	1.0	1.0	1.0	(Feng et al., 1998; Gong et al., 2008)
**AS**	0.4	0.212	0.667	1.3	1.0	1.0	$V_{1/2}$ −6	(Gong et al., 2008)
**LA**	1.0	1.0	1.0	1.0	1.6	1.0	1.0	(Ehrlich et al., 2003; Feng et al., 1998; Li et al., 2001)
**LAA**	1.0	0.68	0.8	1.0	1.6	1.0	1.0	(Caballero et al., 2010; Feng et al., 1998; Li et al., 2001)
**PV**	0.7	0.75	1.0	1.0	2.4	2.0	0.67	(Cha et al., 2005; Datino et al., 2010; Ehrlich et al., 2003)

A summary of conductance scaling factors, G_X, for maximal conductance of ionic current I_X relative to the baseline (RA) cell model and corresponding experimental data sources. Abbreviations are as follows: CT = crista terminalis, BB = Bachmann’s bundle, PM = pectinate muscles, AVR = atrio-ventricular ring, RAA = right atrial appendage, AS = atrial septum, LA = left atrium, LAA = left atrial appendage, PV = pulmonary veins.

Table S2: Rate transitions to propafenone drug-bound states of the Markov chain scheme for I_{Kr}.

	Propafenone
k_A (μ$M^{-1}s^{-1}$)	1.49×10^{3}
l_A (s^{-1})	2.54×10^{-3}
k_I (μ$M^{-1}s^{-1}$)	1.11×10^{2}
l_I (s^{-1})	3.02×10^{-5}
**Open state affinity (μM)**	1.70×10^{0}
**Inactivated state affinity (μM)**	2.72×10^{-1}

Binding (k) and unbinding (l) rates to drug-bound activated (A) and inactivated (I) states corresponding to the drug-bound Markov chain scheme presented in Manuscript Figure 1. Drug affinities for states of type X were determined by computing l_X/k_X.

Table S3: Rate transitions for the guarded receptor model of propafenone binding to I_{Na}.

	**Propafenone**
**k_A (μM^{-1}s^{-1})**	1.49×10^{3}
**l_A (s^{-1})**	2.54×10^{-3}
**k_I (μM^{-1}s^{-1})**	1.11×10^{2}
**l_I (s^{-1})**	3.02×10^{-5}
**Open state affinity (μM)**	1.70×10^{0}
**Inactivated state affinity (μM)**	2.72×10^{-1}

Binding (k) and unbinding (l) rates to drug-bound activated (A), inactivated (I), and resting (R) states corresponding to the guarded receptor model equations. Drug affinities for states of type X were determined by computing l_X/k_X.

Table S4: Secondary effects of disopyramide, quinidine, and propafenone on ion channels.

	**Disopyramide**		**Quinidine**		**Propafenone**	
**Current**	**IC$_{50}$ (μM)**	**Source**	**IC$_{50}$ (μM)**	**Source**	**IC$_{50}$ (μM)**	**Source**
I_{CaL}	1036.7	(Kramer et al., 2013)	14.9	(Zhang and Hancox, 2002)	1.7	(Hancox and Mitcheson, 1997)
I_{to}	20.9	(Hanada et al., 2003)	21.8	(Nenov et al., 1998)	4.8	(Gross and Castle, 1998)
I_{Ks}	88.1		44.0	(Kang et al., 2001)	-	
I_{K1}	-		42.6	(Nenov et al., 1998)	16.8	(Amorós et al., 2013)
I_{NaL}	-		12.0	(Wu et al., 2008)	-	
I_{Kur}	25.0	(Aréchiga et al., 2008)	6.6	(Nenov et al., 1998)	4.4	(Franqueza et al., 1998)

A summary of half maximal inhibitory concentration values (IC$_{50}$) extracted from the literature for disopyramide, quinidine, and propafenone. Block of I_{NaL} (highlighted in red) by quinidine was not included in simulations, as late sodium current is not included in the CNZ model.

Table S5: A summary of 2D and 3D re-entry simulations.

	2D			3D	
	**Meander area (mm^2ms^{-1})**	**Lifespan (s)**		**Dominant frequency (Hz)**	**Lifespan (s)**
**SQT1**	0.35	5.00	**ICs 1**	4.79	10.00
			**ICs 2**	4.99	10.00
**+1 µM DISO**	0.40	5.00	**ICs 1**	N/A	4.93
			**ICs 2**	4.39	10.00
**+2 µM DISO**	0.47	5.00	**ICs 1**	3.99	10.00
			**ICs 2**	3.79	10.00
**+5 µM DISO**	3.18	0.77	**ICs 1**	N/A	7.12
			**ICs 2**	2.99	10.00
**+1 µM QUIN**	0.44	5.00	**ICs 1**	N/A	5.36
			**ICs 2**	3.99	10.00
**+2 µM QUIN**	5.08	0.32	**ICs 1**	N/A	9.13
			**ICs 2**	3.59	10.00
**+5 µM QUIN**	6.66	0.34	**ICs 1**	N/A	6.57
			**ICs 2**	N/A	3.32
**+0.2 µM PROPAF**	2.10	2.82	**ICs 1**	4.99	10.00
			**ICs 2**	4.79	10.00
**+0.5 µM PROPAF**	1.98	3.35	**ICs 1**	N/A	7.21
			**ICs 2**	N/A	5.19
**+0.8 µM PROPAF**	0.75	5.00	**ICs 1**	4.59	10.00
			**ICs 2**	4.79	10.00

A summary of re-entry simulations from idealised 2D homogeneous sheets and the 3D anatomical human atria model, under drug-free SQT1 conditions, and following pharmacological modulation with disopyramide (DISO), quinidine (QUIN), propafenone (PROPAF). Values from clockwise and anticlockwise initial conditions (ICs 1 and ICs 2, respectively) used for 3D simulations are given. Simulations in which re-entry terminated are highlighted in red.

3. Supplementary Videos

Video S1: Re-entrant scroll waves in the WT condition initiated in the 3D anatomical human atria model shown from two views–looking at the RA posterior wall (left) and into the cavities (right). A single scroll wave completes two circuits in the RA before self-terminating after ~0.7 s.

Video S2: Re-entrant scroll waves in the WT-N588K condition initiated in the 3D anatomical human atria model shown from two views–looking at the RA posterior wall (left) and into the cavities (right). Scroll waves meander significantly throughout the atria, before settling into a persistent, anatomical re-entry around the opening of the inferior vena cava which lasts for the remainder of the simulation.

Video S3: Re-entrant scroll waves in the N588K condition initiated in the 3D anatomical human atria model shown from two views–looking at the RA posterior wall (left) and into the cavities (right). Re-entrant wave activity is driven by a persistent, anatomical re-entry around the opening of the inferior vena cava, with occasional existence of multiple wavelets.

Video S4: Re-entrant scroll waves in the SQT1 (WT-N588K) condition under application of 5 μM disopyramide in the 3D anatomical human atria model shown from two views–looking at the RA posterior wall (left) and into the cavities (right). Following application of disopyramide, the wavelength of re-entrant excitations is increased, which leads to termination of re-entry after ~7.1 s.

Video S5: Re-entrant scroll waves in the SQT1 (WT-N588K) condition under application of 2 μM quinidine in the 3D anatomical human atria model shown from two views–looking at the RA posterior wall (left) and into the cavities (right). Following application of quinidine, the wavelength of re-entrant excitations is increased, which leads to termination of re-entry after ~9.1 s.

Video S6: Re-entrant scroll waves in the SQT1 (WT-N588K) condition under application of 0.5 μM propafenone in the 3D anatomical human atria model shown from two views–looking at the RA posterior wall (left) and into the cavities (right). Following application of propafenone, secondary waves are induced which destabilise the re-entrant circuit, leading to termination of re-entry at ~7.2 s.

Video S7: Initiation and conduction of spiral waves in a 2D idealised geometry in drug-free SQT1 (top left), SQT1 + K^+ channel (I_{Kr}) block (top right), SQT1 + Na^+ channel (I_{Na}) block (bottom left), and SQT1 + K^+ and Na^+ channel (I_{Kr} + I_{Na}) block (bottom right) conditions. Re-entry terminates only under K^+ and Na^+ channel block conditions.

References

Amorós, I., Dolz-Gaitón, P., Gómez, R., Matamoros, M., Barana, A., de la Fuente, M. G., et al. (2013). Propafenone blocks human cardiac Kir2.x channels by decreasing the negative electrostatic charge in the cytoplasmic pore. *Biochem. Pharmacol.* 86, 267–278. doi:10.1016/j.bcp.2013.04.023.

Aréchiga, I. A., Barrio-Echavarria, G. F., Rodríguez-Menchaca, A. A., Moreno-Galindo, E. G., Decher, N., Tristani-Firouzi, M., et al. (2008). Kv1.5 Open Channel Block by the Antiarrhythmic Drug Disopyramide: Molecular Determinants of Block. *J. Pharmacol. Sci.* 108, 49–55. doi:10.1254/jphs.08084FP.

Burashnikov, A., Mannava, S., and Antzelevitch, C. (2004). Transmembrane action potential heterogeneity in the canine isolated arterially perfused right atrium: effect of IKr and IKur/Ito block. *Am. J. Physiol. - Heart Circ. Physiol.* 286, H2393–H2400. doi:10.1152/ajpheart.01242.2003.

Caballero, R., de la Fuente, M. G., Gómez, R., Barana, A., Amorós, I., Dolz-Gaitón, P., et al. (2010). In Humans, Chronic Atrial Fibrillation Decreases the Transient Outward Current and Ultrarapid Component of the Delayed Rectifier Current Differentially on Each Atria and Increases the Slow Component of the Delayed Rectifier Current in Both. *J. Am. Coll. Cardiol.* 55, 2346–2354. doi:10.1016/j.jacc.2010.02.028.

Cha, T.-J., Ehrlich, J. R., Zhang, L., Chartier, D., Leung, T. K., and Nattel, S. (2005). Atrial Tachycardia Remodeling of Pulmonary Vein Cardiomyocytes. *Circulation* 111, 728–735. doi:10.1161/01.CIR.0000155240.05251.D0.

Courtemanche, M., Ramirez, R. J., and Nattel, S. (1998). Ionic mechanisms underlying human atrial action potential properties: insights from a mathematical model. *Am. J. Physiol. - Heart Circ. Physiol.* 275, H301–H321.

Datino, T., Macle, L., Qi, X.-Y., Maguy, A., Comtois, P., Chartier, D., et al. (2010). Mechanisms by Which Adenosine Restores Conduction in Dormant Canine Pulmonary Veins. *Circulation* 121, 963–972. doi:10.1161/CIRCULATIONAHA.109.893107.

Ehrlich, J. R., Cha, T.-J., Zhang, L., Chartier, D., Melnyk, P., Hohnloser, S. H., et al. (2003). Cellular electrophysiology of canine pulmonary vein cardiomyocytes: action potential and ionic current properties. *J. Physiol.* 551, 801–813. doi:10.1113/jphysiol.2003.046417.

Feng, J., Yue, L., Wang, Z., and Nattel, S. (1998). Ionic Mechanisms of Regional Action Potential Heterogeneity in the Canine Right Atrium. *Circ. Res.* 83, 541–551. doi:10.1161/01.RES.83.5.541.

Franqueza, L., Valenzuela, C., Delpón, E., Longobardo, M., Caballero, R., and Tamargo, J. (1998). Effects of propafenone and 5-hydroxy-propafenone on hKv1.5 channels. *Br. J. Pharmacol.* 125, 969–978. doi:10.1038/sj.bjp.0702129.

Gong, D., Zhang, Y., Cai, B., Meng, Q., Jiang, S., Li, X., et al. (2008). Characterization and comparison of Na+, K+ and Ca2+ currents between myocytes from human atrial right

appendage and atrial septum. *Cell. Physiol. Biochem. Int. J. Exp. Cell. Physiol. Biochem. Pharmacol.* 21, 385–394. doi:10.1159/000129631.

Gross, G. J., and Castle, N. A. (1998). Propafenone Inhibition of Human Atrial Myocyte Repolarizing Currents. *J. Mol. Cell. Cardiol.* 30, 783–793. doi:10.1006/jmcc.1998.0643.

Hanada, E., Ohtani, H., Hirota, M., Uemura, N., Nakaya, H., Kotaki, H., et al. (2003). Inhibitory effect of erythromycin on potassium currents in rat ventricular myocytes in comparison with disopyramide. *J. Pharm. Pharmacol.* 55, 995–1002. doi:10.1211/0022357021459.

Hancox, J. C., and Mitcheson, J. S. (1997). Inhibition of L-type calcium current by propafenone in single myocytes isolated from the rabbit atrioventricular node. *Br. J. Pharmacol.* 121, 7–14. doi:10.1038/sj.bjp.0701086.

Kang, J., Chen, X.-L., Wang, L., and Rampe, D. (2001). Interactions of the Antimalarial Drug Mefloquine with the Human Cardiac Potassium Channels KvLQT1/minK and HERG. *J. Pharmacol. Exp. Ther.* 299, 290–296.

Kramer, J., Obejero-Paz, C. A., Myatt, G., Kuryshev, Y. A., Bruening-Wright, A., Verducci, J. S., et al. (2013). MICE Models: Superior to the HERG Model in Predicting Torsade de Pointes. *Sci. Rep.* 3. doi:10.1038/srep02100.

Li, D., Zhang, L., Kneller, J., and Nattel, S. (2001). Potential Ionic Mechanism for Repolarization Differences Between Canine Right and Left Atrium. *Circ. Res.* 88, 1168–1175. doi:10.1161/hh1101.091266.

Nenov, N. I., Crumb, W. J., Pigott, J. D., Harrison, L. H., and Clarkson, C. W. (1998). Quinidine Interactions With Human Atrial Potassium Channels. *Circ. Res.* 83, 1224–1231. doi:10.1161/01.RES.83.12.1224.

Ni, H., Whittaker, D. G., Wang, W., Giles, W. R., Narayan, S. M., and Zhang, H. (2017). Synergistic anti-arrhythmic effects in human atria with combined use of sodium blockers and acacetin. *Front. Physiol.* 8. doi:10.3389/fphys.2017.00946.

O'Hara, T., Virág, L., Varró, A., and Rudy, Y. (2011). Simulation of the Undiseased Human Cardiac Ventricular Action Potential: Model Formulation and Experimental Validation. *PLoS Comput. Biol.* 7. doi:10.1371/journal.pcbi.1002061.

Tusscher, K. H. W. J. ten, and Panfilov, A. V. (2006). Alternans and spiral breakup in a human ventricular tissue model. *Am. J. Physiol. - Heart Circ. Physiol.* 291, H1088–H1100. doi:10.1152/ajpheart.00109.2006.

Wu, L., Guo, D., Li, H., Hackett, J., Yan, G.-X., Jiao, Z., et al. (2008). Role of late sodium current in modulating the proarrhythmic and antiarrhythmic effects of quinidine. *Heart Rhythm* 5, 1726–1734. doi:10.1016/j.hrthm.2008.09.008.

Yu, W. C., Lee, S. H., Tai, C. T., Tsai, C. F., Hsieh, M. H., Chen, C. C., et al. (1999). Reversal of atrial electrical remodeling following cardioversion of long-standing atrial fibrillation in man. *Cardiovasc. Res.* 42, 470–476.

Zhang, Y., and Hancox, J. (2002). Mode-dependent inhibition by quinidine of Na+–Ca2+ exchanger current from guinea-pig isolated ventricular myocytes. *Clin. Exp. Pharmacol. Physiol.* 29, 777–781. doi:10.1046/j.1440-1681.2002.03731.x.
